# Clays for Low‐Carbon Cements: Overview, Progress, and Challenges

**DOI:** 10.1002/gch2.202500496

**Published:** 2026-01-27

**Authors:** Imane Koufany, Isabel Santacruz, Angeles G. De la Torre, María D. Rodríguez‐Ruiz, Miguel A. G. Aranda

**Affiliations:** ^1^ Departamento de Química Inorgánica Cristalografía y Mineralogía University of Malaga Malaga Spain; ^2^ Instituto Universitario de Materiales y Nanotecnología IMANA University of Malaga Malaga Spain

**Keywords:** artificial pozzolans, compressive strengths, low CO_2_ footprint, mechanical activation, R^3^ test, SCMs, thermal activation

## Abstract

The replacement of Portland clinker with supplementary cementitious materials is a key approach to reducing the embodied carbon content of concretes. In this context, a widely studied family is the “limestone calcined clay cements, LC^3^.” Within this eco‐friendly family of materials, one composition is gaining popularity, LC^3^‐50, a blend of ~50% of Portland clinker, 30% of activated clay, 15% of limestone and 5% of gypsum. This interest is due to a ~40% reduction of CO_2_ emissions compared to Portland clinker, together with high compressive strengths after 7 days and very good durability against chloride and sulfate attacks. However, limitations still exist, such as low strengths at 1 day, workability loss during the first 2 h and reduced carbonation resistance. These drawbacks are being overcome with tailored admixtures and curing approaches. Here, after introducing low‐carbon cements, pozzolans, and pozzolanic reactions, as well as phyllosilicate minerals, attention is given to recent progress in thermal and mechanical, aka mechanochemical, activations. Then, general correlations are established to assist in predicting compressive strength. This work concludes by highlighting the challenges that must be overcome for the widespread adoption of these classic rocks processed to yield advanced materials with the highest possible pozzolanic reactivity.

## Graphical abstract

This review provides a critical overview of recent developments of activated clays to function as artificial pozzolans. After a thorough description of the clays that yield good pozzolanic performances, selected examples are given on thermal and mechanical activations. Chiefly, general correlations are extracted from reported data despite its scattered nature. Finally, key challenges are identified and discussed.

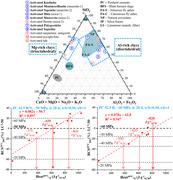

## 1 Brief Introduction to the Need and Nature of Low‐Carbon Cements and Concretes

Cement production is essential for any society wishing to have healthy, comfortable, safe, and secure environments. In addition, it bolsters societal progress. This, however, comes at a price. Cement manufacturing, with a worldwide cement production of 4.1 gigatonnes (Gt) in 2023 [1], is associated with huge CO_2_ emissions (i.e., 1.57 Gt in 2023) [2] and high energy consumption. This is related to the manufacturing of Portland cement (PC), which is fabricated by grinding Portland clinker with a small amount of calcium sulfate source (and in many cases calcium carbonate). In turn, Portland clinker is obtained by heating a mixture of limestone (LS) and clay at 1450°C to obtain a multiphase material with an approximate composition of 55–75 wt.% of alite (Ca_3_SiO_5_ with elemental impurities), 10–20 wt.% of belite (Ca_2_SiO_4_ with elemental impurities), ~10 wt.% of ferrite (Ca_4_Al_2_Fe_2_O_10_) and ~5–10 wt.% of tricalcium aluminate (Ca_3_Al_2_O_6_) [3]. During grey Portland clinker fabrication, ∼0.85 tonnes of CO_2_ are released per tonne of product [4], mainly due to the decarbonation of LS and fuel combustion. These CO_2_ emissions account for ~8% of all anthropogenic CO_2_ [5], and thus, lowering the carbon footprint of cement production is an imperative societal need [6, 7].

Several organizations including the World Green Building Council and the Global Cement and Concrete Association, have set ambitious targets for the building sector of a 50% reduction of emissions by 2030 and net‐zero emissions by 2050. To achieve the carbon neutrality target for 2050, the European Cement Association has developed a roadmap built on five key pillars known as the 5C strategies [8] based on clinker, cement, concrete, construction, and carbonation approaches. A widespread substitution of (high‐CO_2_ footprint) Portland clinker with (low‐CO_2_ footprint) supplementary cementitious materials (SCMs) is currently the most advanced and available strategy for reducing the carbon footprint of cements [9, 10, 11]. According to the European standard EN‐UNE 197‐5 [12], cements blended with SCMs are classified according to their composition. For instance, CEM II/C‐M composition establishes contents of Portland clinker and SCM of 50–64 wt.% and 36–50 wt.%, respectively, where the amount of limestone cannot exceed 20 wt.%, and 0–5 wt.% of minority components [12]. LC^3^ (Limestone Calcined Clay Cements) follow this classification in Europe (viz. CEM II/C‐M (Q‐L(L))) [13, 14, 15]. In LC^3^‐50 cements, 50 wt.% of the Portland clinker is replaced by activated clay (~30 wt.%), limestone (~15 wt.%), and gypsum/calcium sulfate(s) (~5 wt.%); this allows reductions in CO_2_ emissions of 35%–40% compared to PC type I [14].

Moreover, the final products are mortars and chiefly, concretes. Concretes are fabricated by mixing PC, water, sand, and coarse aggregates and adding the adequate admixtures (chemicals) to tailor their properties/performances. Concrete is the most widely used commodity, after fresh water, so its price is key. After a careful survey in Spain/Europe by the authors, Table 1 reports the prices of the most widely used inorganic materials for concrete production including the currently employed SCMs. Emerging SCMs [10], under investigation but not on the market, are not included in Table 1 as their prices are unknown to the authors.

**TABLE 1:** Prices of raw materials for concrete production in Spain/Europe in 2025.

Raw materials	Price [€ ton^−1^]	Notes
CEM I (grey)	130–140	^a^, less available with time
CEM I (white)	220	For niche applications
CEM II	90–100	—
Gypsum (GYP)	25–40	—
Limestone (LS)	30–40	—
Granulated Blast Furnace Slag (GBFS)	100–200	Prices varies depending on performances
Fly Ash (FA)	50–100	^b^
Natural Pozzolan (NP)	50–100	^b^
Calcined Clay (CC)	50–100	^b^, ^c^
Metakaolin (MK)	400–600	^d^, white MK > 800 €/ton, when available
Silica Fume (SF)	400–600	^d^
Natural Sand	10–20	—
Natural Aggregate	10–20	—

^a^ Price refers to PC 52.5 R, with a sharp increase of about 40 €/ton in the last 2 years.

^b^ FA/NP/CC have low availability in Europe; therefore, their prices are quite unpredictable, i.e., difficult to fix. They depend on local availabilities and time‐dependent procurement approaches.

^c^ There is still no market for mechanically‐activated clays in Europe.

^d^ MK and SF are used for high‐value applications such as ultra‐high‐performance concretes.

Some PC‐SCMs blends, such as activated clays, face the same two key challenges: (1) low hydration rate at early ages, i.e., 1–2 days, meaning low compressive strengths; and (2) poor flowability after mixing and problems to maintain the flow of the ready‐mix concrete during the first 1–2 h. Therefore, the addition of admixtures are key to be able to widely adopt low‐carbon cements for most applications [16, 17, 18]. Thus, Table 2 reports the prices of selected admixtures (organic chemicals) that are being used to manufacture concretes and to address the challenges stated above.

**TABLE 2:** Prices of selected admixtures for concrete production in Spain/Europe in 2025.

Admixture	Price [€ ton^−1^]	Notes
PCE‐standard	1100–1500	^a^, for ~25‐30% active matter content
PCE‐advanced	1400–1800	^b^
MonoEthylene Glycol (MEG)	700–900	Used as grinding aid in PC manufacturing
DiEthylene Glycol (DEG)	900–1100	Used as grinding aid in PC manufacturing
Triisopropanolamine (TIPA)	1800–2200	^c^, Highly fluctuating prices
Triethanolamine (TEA)	1500–1800	^c^
Master X‐Seed 100	1000–1200	^d^
Master X‐Seed STE	1500–1800	^e^

^a^ Standard PolyCarboxylate Ether (PCE) superplasticizer (SP). PCE‐SPs with lower amounts of active matter usually have lower prices, and vice versa.

^b^ State‐of‐the‐art PCE‐SPs tailored for low‐carbon cements addressing challenging field conditions (f.i., maintaining the slump/flow in the first ~90 min).

^c^ Tertiary alkanolamine used as clinker grinding aid and also as strength enhancing admixture (SEA).

^d^ First generation C‐S‐H gel nucleation seeding admixture working as accelerator and SEA in concretes.

^e^ State‐of‐the‐art C‐S‐H gel nucleation seeding admixture tailored for low‐carbon cements.

In addition to price, the CO_2_‐footprint is a key figure of merit in the production of low‐carbon concretes. Therefore, Table 3 displays embodied carbon contents [kg of equivalent CO_2_ by kg of product] for selected inorganic materials employed for the fabrication of low‐carbon cements and concretes. The values are approximate and they cannot accurately account for transportation and grinding, which have local‐dependent CO_2_‐footprints. In any case, a LC^3^‐50 binder has an approximate embodied carbon of ~0.85 × 0.50 + 0.35 × 0.30 + 0.003 × 0.15 + 0.002 × 0.05 or 0.53 kg CO_2_‐e. This means about 38% reduced CO_2_ emissions when compared to type‐I PC.

**TABLE 3:** Embodied carbon of raw materials for concrete production.

Raw materials	Embodied carbon [kg CO_2_‐e kg^−1^]	Notes	Refs.
PC (type‐I)	0.840–0.850	Footprint by using common raw materials	[15, 19]
Gypsum (GYP)	0.002		[19]
Limestone (LS)	0.002–0.004		[15, 19, 20]
Granulated Blast Furnace Slag (GBFS)	0.080–0.183	Grinding, transport and batching	[21, 22]
Fly Ash (FA)	0.004–0.170		[15, 19]
Natural Pozzolan (NP)	0.027	Approximate value that includes transport. Grinding excluded	[23]
Calcined Clay (CC)	0.250–0.393	A value of 0.196 kg CO_2_‐e/kg was reported for Kiln industrial trial	[24, 25]
Metakaolin (MK)	0.372–0.400		[25, 26]
Silica Fume (SF)	0.003–0.024	Transportation (100 km) is included	[19, 27]
Natural Sand	0.003–0.011	[22] Sand at batching plant	[22, 26]
Natural Aggregate	0.008–0.011		[22, 26]

It can be argued that the quantities of admixtures required to prepare low‐carbon concretes, with competitive performances, are larger than those needed for fabricating standard concretes. This is indeed the case and therefore, Table 4 displays embodied carbon contents for selected admixtures. By assuming a requirement of 1% of PCE‐SP (by the weight of binder), to have appropriate rheological properties, and 2% of C‐S‐H gel nucleation seeding (as a SEA representative), to get relatively good compressive strengths at 1 day of hydration, the approximate embodied carbon for a LC^3^‐50 binder would be 0.53 + 0.69 × 0.01 + 0.69 × 0.02 or 0.55 kg CO_2_‐e. This means around 35% reduced CO_2_ emissions when compared to type‐I PC without any admixture requirement.

**TABLE 4:** Embodied carbon of selected admixtures for concrete production.

Additive	Embodied carbon [kg CO_2_‐e kg^−1^]	Notes	Refs.
PCE‐s	0.750–1.180		[15, 25]
PCE‐adv	0.689		[28]
MonoEthylene Glycol (MEG)	1.2–1.4^a^ 5.3‐7.5^b^ 0.4^c^	The value depends on the synthesis procedure: ^a^ fossil based‐route; ^b^ gasification process; ^c^ biomass gasification	[29]
Triisopropanolamine (TIPA)	0.418		[30]
Triethanolamine (TEA)	3.033		[31]
Master X‐Seed 100	0.726		[32]
Master X‐Seed STE	0.721		[33]

To end this section, it is noted that the production of the most currently used SCMs (GBFS, FA, and SF) is declining due to the decarbonizing strategies of the industries concerned. Therefore, their supply is currently limited and expected to be much lower in the near future. In this context, activated clays (and natural pozzolans, if they are locally available with enough reactivity) are emerging as the most promising alternative, as their availability is guaranteed and they help substantially reduce the footprint of the final products: cements and concretes. The focus here is on clays already in use (and to be used) as SCMs. After a very brief introduction to the pozzolanic materials and reactions and to the crystallochemistry of clays, the attention turns to the activation (thermal and mechanical) of different families of clays to be effectively employed as SCMs. Then, this work concentrates on the correlation between the properties of activated clays and the compressive strength performances of the corresponding mortars. The final part of the manuscript focusses on the challenges followed by some concluding remarks.

## 2 Significance and Novelty of this Work

It is acknowledged that there are many reviews dealing with LC^3^ cement [13, 14, 15, 34, 35] and activated calcined clays to be used as SCMs [36, 37, 38, 39]. Moreover, it is very important to note here the ten whitepaper of RILEM Technical Committee 282‐CCL “Calcined Clays as Supplementary Cementitious Materials” which has been summarized very well in the closing letter [40]. However, it is also noted that most of the review works focused on thermally activated kaolinitic clays. This work moves forward by comparing thermal and mechanical activations for several types of clays beyond the common ones (i.e., kaolinite, smectite, and illite).

This work is not just a compilation of literature findings, but the novelty and significance are due to: (1) analysis of cost and environmental impact; (2) comparison of activation methods, i.e., thermal versus mechanical; (3) a unified structural description of clays; and (4) Chiefly, general correlations between the pozzolanic reactivities (as measured by the R^3^ method) and mechanical strengths which are extracted from the bibliography despite its scattered nature. Moreover, most of the reported works used PC 42.5 type cements with less investigations using PC 52.5 cements. A detailed comparison is given in section 7, which indicates that PC42.5 cements appear to be more suitable for producing LC^3^‐type binders, if the target is mechanical strengths of 50 MPa, or smaller, at 28 days. Finally, key challenges are identified and discussed.

## 3 Brief Introduction to the Pozzolanic Materials, Methods of Characterization and Reactions

The use of calcined clays as artificial pozzolans dates back to as early as 1787, in the seminal work of Chaptal [41]. A pozzolan is defined in the ASTM C125 standard as a siliceous or siliceous and aluminous non‐cementitious material that reacts with Ca(OH)_2_ to form compounds with cementitious properties. SCMs used in blends can be classified as artificial or natural pozzolans, all have in common high amorphous SiO_2_ (and in some cases Al_2_O_3_ and/or Fe_2_O_3_) content(s). On the one hand, artificial pozzolans are usually industrial by‐products [10] such as FA, SF, GBFS (partly hydraulic), or biomass ashes [42, 43, 44]. On the other hand, natural pozzolans [11, 45] usually show volcanic origin, such as volcanic ash and/or glass, pumice, and perlite [45, 46, 47], or meteorized materials such as some zeolites (i.e., trass); but there may also be a biogenic origin (for instance diatomite) [45]. Activated/calcined clays are located somewhere in between, as they can be considered “treated” natural pozzolans [14, 48, 49].

Activated clays are mainly based on kaolinite‐ (1:1 phyllosilicate), smectite‐ and illite‐ (2:1 phyllosilicate). Raw clays (before activation) will be treated in the next section. Here, it is introduced that clays can be activated in three ways: (1) thermally, (2) mechanically [50], and (3) chemically (acid treatment) [51, 52, 53]. It is noted that a very recent paper has activated kaolinite, halloysite, and montmorillonite using all three approaches [54]. The activation methodologies will be discussed in sections 5 and 6.

The pozzolanic reactions have slower kinetics than that of the hydration of alite, the main component of PC. The cementitious properties of the resulting hydrates permit a relatively large replacement of PC by pozzolanic materials. However, not all pozzolanic materials can be used in cements [10], as reactivity (i.e., ultimate degree of reaction) and kinetic (i.e., reaction rate), together with certain chemical (composition) and physical (f.i., specific surface area (SSA) and particle size distribution) properties are needed to prepare competitive blends. In addition, material availability and fitting standards are presently also essential characteristics [10] because prescriptive, and not performance‐based standards, are still in practice. For instance, ASTM C618‐23 focuses on FA, raw and calcined natural pozzolans; ASTM C989/C989M‐22 deals with GBFS; and ASTM C1240‐20 treats SF. Moreover, ASTM C1697‐21 focuses on mixtures of two or three ASTM‐compliant SCMs.

There are many tests to characterize the pozzolanic activity of candidates for SCMs [39]. A more in‐depth presentation is beyond the scope of this manuscript, so here we initially mention just three: (i) modified Chapelle test, (ii) Frattini test, and (iii) Indian Standard lime reactivity test [39, 55, 56]. As the most important parameter at stake is the compressive strengths, currently, ASTM assesses SCM reactivity through the strength activity index (SAI) in C311 standard [57]. The SAI assay determines the mortar compressive strength at 7 and 28 days at a 20% replacement by mass and at a varying water‐to‐binder ratio (w/b) to meet a flow of ±5% of the neat cement control mixture at a w/b value of 0.484. A material passes the test if the mortar compressive strength is at least 75% that of the control at 7 or 28 days [58]. The SAI test is a physical test used to determine material compliance to ASTM C618 [58]. The corresponding European standard measures the compressive strength of mortars at 28 and 90 days but with a 25% replacement by mass [59], which minimizes the filler effects. Compressive strength of mortars must be 75% of the control at 28 days as well as 85% at 90 days. Unlike ASTM C618, there is no flow requirement, and water content is held constant. It is noted that RILEM commonly uses a 30% replacement of PC by SCMs in order to study the pozzolanic properties of SCMs including the SAI values [60]. There are many papers reporting the problems of using 80%PC‐20%SCMs as some non‐pozzolanic materials pass the ASTM‐C311 assay because the filler effect [55, 61, 62].

The SAI test is done at 20°C and it takes 28 (and/or) 90 days. In order to have a faster characterization of the pozzolanic activity, a new rapid, relevant, and reliable (R^3^) test, was developed at 40°C for calcined clays [63, 64], and tested for other SCMs by RILEM TC 267‐TRM [60], with good correlation between the released heat, as measured by isothermal calorimetry, and compressive strengths of mortars of the corresponding blends. The method was then standardized as ASTM C1897, including the continuous heat released during seven days and the bound water at seven days [65]. The standard gives prescribed amounts of Ca(OH)_2_, CaCO_3_, KOH, K_2_SO_4_, and distilled H_2_O, and therefore, it is not affected by the (variable) hydration kinetics of PCs.

To end this section, we sketch the pozzolanic reactions. The most common one is given in reaction (1) [66] where amorphous SiO_2_ (from the natural pozzolan or the activated clay) reacts with Ca(OH)_2_ (from the hydration of PC) to yield additional cementitious material: nanocrystalline calcium silicate hydrate also known as C‐S‐H gel [67, 68]. It is noted that the stoichiometry given in reaction (1) for C‐S‐H gel is approximate, as it may contain variable amounts of aluminium and other elements, and the water content is also variable. However, the discussion of C‐(A)‐S‐H features is beyond the scope of this study.

(1) $$\mathrm{Si}O_{2}\;+\;1.5\mathrm{Ca}{\left(\mathrm{OH}\right)}_{2}\;+\;2.5H_{2}O\;\to \;Ca_{1.5}\mathrm{Si}O_{3.5}{\left(H_{2}O\right)}_{4}$$

Some authors have studied the pozzolanic reactions for PC‐blends with thermally activated Al‐rich clays [69, 70, 71]. Thus far, the majority have centred on metakaolin, the ideal composition of Al_2_Si_2_O_7_, as the archetype for metaclays. The pozzolanic reaction of metakaolin can be understood as the result of the combination of two cementitious products, silicon‐rich and aluminium‐rich hydrates. In the absence of sulfate and carbonate anions, metakaolin reacts with Ca(OH)_2_ in the presence of water to yield C‐A‐S‐H gel, stratlingite and AFm‐type calcium aluminate hydrate phase(s), see reaction (2). The stoichiometry of this C‐A‐S‐H gel is variable and it shows lower Ca/Si [72] and higher Al/Si ratios than in neat‐PC [73]. However, this hydration environment is not the common situation in LC^3^ binders, where there are limited amounts of sulfates and plenty of carbonates. If the hydration of metakaolin takes place at a time where there are still available sulfates, the main reaction is (3). However, the most common situation is that the hydration takes place when sulfates are depleted and there are plenty of carbonates, see reaction (4) for hemicarbonaluminate formation.

(2) $$\begin{array}{ccc} Al_{2}Si_{2}O_{7} & & +(6.2-3.1a)\mathrm{Ca}{\left(\mathrm{OH}\right)}_{2}+(12.2-4.6a)H_{2}O\\ & & \to (2-a)Ca_{1.5}Al_{0.2}\mathrm{Si}O_{3.8}{\left(H_{2}O\right)}_{4.0}+aCa_{2}Al_{2}\mathrm{Si}O_{7}\cdot 8H_{2}O\\ & & +(0.8-0.9a)Ca_{4}Al_{2}{\left(\mathrm{OH}\right)}_{14}\cdot 6H_{2}O \end{array}$$

(3) $$\begin{array}{ccc} Al_{2}Si_{2}O_{7} & & +5.4\mathrm{Ca}{\left(\mathrm{OH}\right)}_{2}+2.4\mathrm{CaS}O_{4}\cdot 2H_{2}O\\ & & +23.4H_{2}O\to 2Ca_{1.5}Al_{0.2}\mathrm{Si}O_{3.8}{\left(H_{2}O\right)}_{4.0}\\ & & +0.8Ca_{6}Al_{2}{\left(SO_{4}\right)}_{3}{\left(\mathrm{OH}\right)}_{12}\cdot 26H_{2}O \end{array}$$

(4) $$\begin{array}{ccc} Al_{2}Si_{2}O_{7} & & +5.8\mathrm{Ca}{\left(\mathrm{OH}\right)}_{2}+0.4\mathrm{CaC}O_{3}\\ & & +11.8H_{2}O\to 2Ca_{1.5}Al_{0.2}\mathrm{Si}O_{3.8}{\left(H_{2}O\right)}_{4.0}\\ & & +0.8Ca_{4}Al_{2}{\left(\mathrm{OH}\right)}_{13}{\left(CO_{3}\right)}_{0.5}{\left(H_{2}O\right)}_{5.5} \end{array}$$

## 4 Raw Clays for Low‐Carbon Cements

The definitions of clays and clay minerals are fundamental for researchers trying to understand and use these materials across different scientific disciplines. Clay minerals have been characterized in numerous ways depending on their specific applications [74, 75, 76], with definitions established according to the particular requirements of different fields such as geology, materials sciences, and engineering [39, 77, 78, 79, 80]. According to the joint nomenclature committees of the Association Internationale pour l'Étude des Argiles (AIPEA) and the Clay Minerals Society (CMS), *clay* is defined as “a naturally occurring material composed primarily of fine‐grain minerals, which is generally plastic at appropriate water contents and will harden when dried or fired” [81]. *Clay minerals* can be defined as “phyllosilicate minerals which impart plasticity to clay and which harden upon drying or firing” [82]. This distinction is fundamental as it separates the material (a clay is a rock from a geological perspective) from its mineral components (clay minerals), establishing a clear framework for scientific understanding and practical applications. Consequently, clays (bentonites, marls, lutites, etc.) principally comprise phyllosilicates, among other minerals. As an example: ● *bentonites* are rocks consisting mainly of smectite minerals; ● *smectite* is a family of phyllosilicates with a given crystal structure, see below; and ● *montmorillonite* is a mineral within the smectite group (i.e., aluminium‐rich).

**FIGURE 1:**
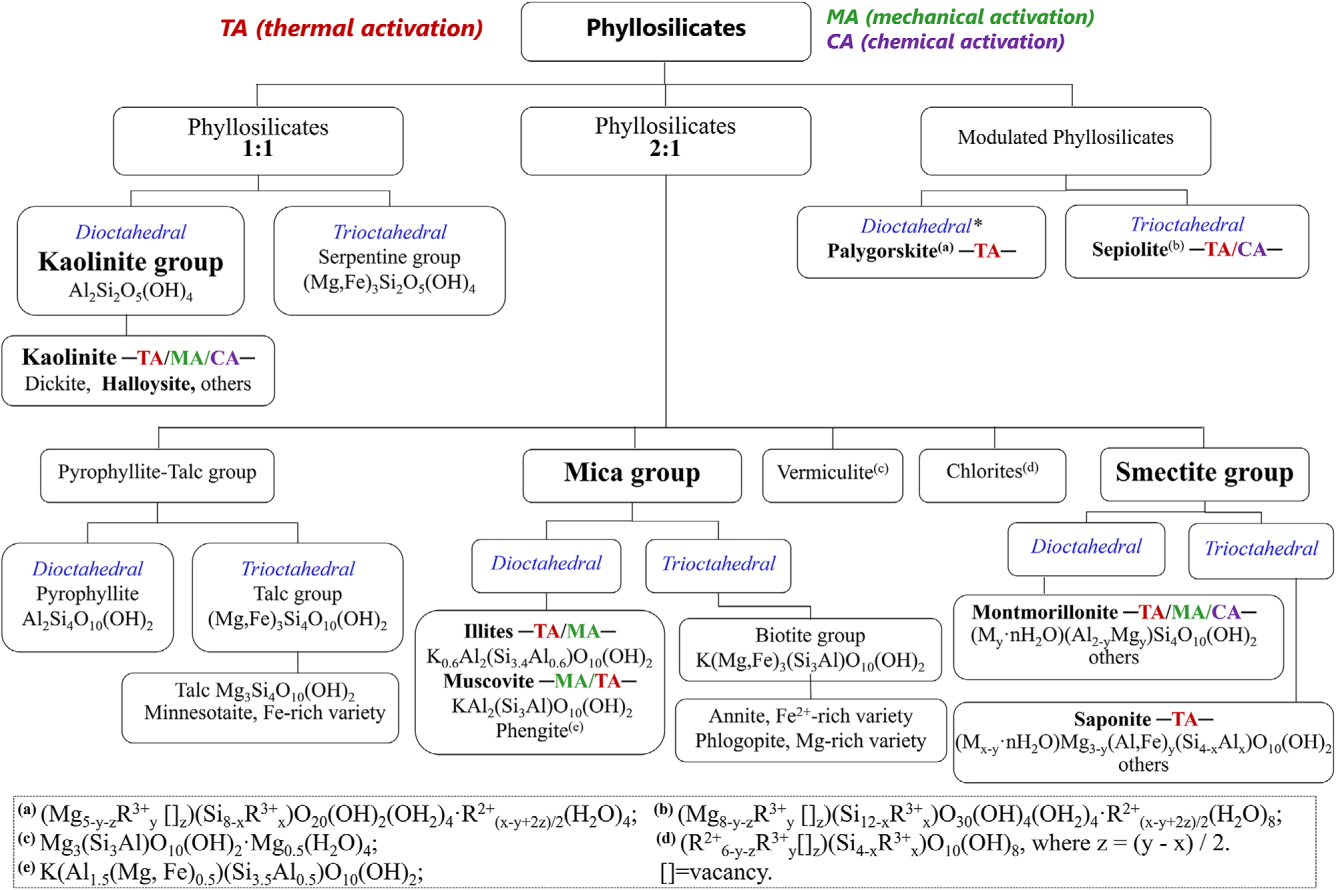
Classification of clay minerals following Bailey (1980) [83] and Rieder et al. (1998) [84]. The clay minerals that have been proved as SCMs are highlighted in bold (i.e., thermal, mechanical, or chemical activations). *Palygorskite has an intermediate tri‐dioctahedral character, approximately 80% of the octahedral sheet is occupied.

Phyllosilicates are a major group of silicates characterized by layered crystal structures and the presence of hydroxyl groups (─OH) and/or water molecules within their structures [83]. Figure 1 presents the chemical classification of phyllosilicates based on their crystal structures. The bold clay minerals include recognized SCMs (kaolinite, smectite, and illite) and emerging candidates (palygorskite, sepiolite, or muscovite), which constitute the focus of this review. Other clay minerals are also given in Figure 1, but their pozzolanic activities after calcination/activation have not been established, so far. Thermal activation has received much more attention than mechanical, but there is a growing interest in this second approach.

**FIGURE 2:**
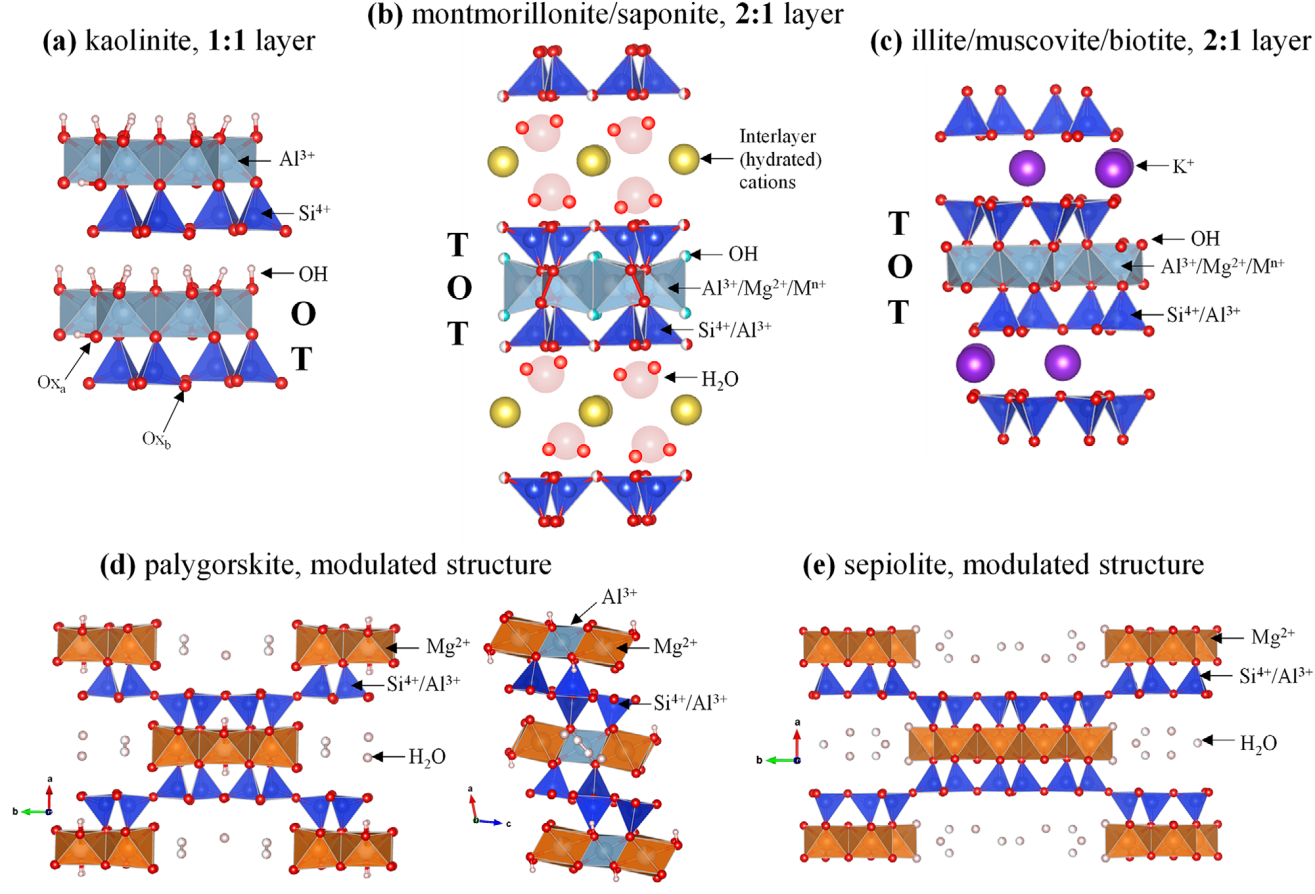
Crystal structures of phyllosilicate minerals with proven pozzolanic activity after activation: (a) kaolinite; (b) smectites; c) micas; (d) palygorskites; and (e) sepiolites.

The structural classification of phyllosilicates is based on the arrangement of tetrahedral and octahedral sheets, which form the fundamental “building blocks” on their crystal structure, see Figure 2 [85, 86, 87]. The tetrahedral sheets consist of silicon‐oxygen tetrahedra linked through three shared basal oxygens (Ox_b_ in Figure 2a), forming hexagonal 2D tetrahedral network with Si^4+^ as the dominant cation, accompanied by Al^3+^ (Figure 2a) [37, 83, 87]. In contrast, octahedral sheets are formed by edge‐sharing octahedra containing Al^3+^, Fe^3+^, Mg^2+^, and/or Fe^2+^ cations coordinated to oxygens (Ox_a_) and hydroxyl groups, see Figure 2a [37, 83, 87]. The octahedral site occupancy determines the mineral classification as trioctahedral or dioctahedral. In trioctahedral phyllosilicates, almost all positions are occupied, predominantly by Mg^2+^, whereas dioctahedral phyllosilicates exhibit approximately two‐thirds occupancy, predominantly by Al^3+^ [83]. The 1:1 layer arrangement (TO layers) consists of alternating tetrahedral and octahedral sheets, while the 2:1 structure (TOT layers) comprises one octahedral sheet sandwiched between two tetrahedral sheets [37, 83, 87].

The 1:1 layer clay minerals include two groups: the kaolin and the serpentine, see Figure 1. The kaolin group, essential for LC^3^ applications [88], has a Al_2_Si_2_O_5_(OH)_4_ composition before activation. This group encompasses kaolinite, dickite, nacrite and halloysite polytypes, which are differentiated by octahedral vacancy positions in adjacent layers [89, 90].

The 2:1 layer clay minerals include five main groups, see Figure 1. For these phases, except the talc‐pyrophyllite group, isomorphous substitutions in the tetrahedral and octahedral sheets generate variable charge deficits which are compensated by monovalent and divalent cations, and in some cases water, in the interlayer region. Here, we focus on the two most important groups for low‐carbon cements: smectites and micas. Smectites are characterized by negative layer charges between 0.2 and 0.6 per formula unit, (Na_0.4_·nH_2_O)(Al_1.8_Mg_0.2_[])Si_4_O_10_(OH)_2_ for sodium montmorillonite, resulting from isomorphic substitutions in the octahedral (and also tetrahedral) sheets. The negative layer charge is compensated by interlayer cations, such as Na^+^, K^+^, Mg^2+^, or Ca^2+^, which are hydrated and exchangeable, see Figure 2b [91]. The corresponding minerals exhibit swelling behaviour, with interplanar spacings ranging from 12.0 to 15.4 Å depending on cation type and hydration state. Montmorillonite, see Figure 2b, is the most common dioctahedral smectite [87] and saponite is the most abundant trioctahedral one. Saponites show MgO contents from 23% to 33%, reflecting their compositional variability [92].

Micas (including muscovite, illite, and biotite) are 2:1 phyllosilicates but they have higher charge density and are not expandable, see Figure 2c [84, 93]. Muscovite exhibits a dioctahedral character with the idealized formula KAl_2_[](Si_3_Al)O_10_(OH)_2_, while biotite displays a trioctahedral nature with ~K(MgFe_2_)(Si_3_Al)O_10_(OH)_2_, being an intermediate member in the solid solution between phlogopite (Mg‐rich) and annite (Fe‐rich) end‐members [93]. Muscovite and biotite are classified as true micas with layer charge x = −1.0 per formula unit, compensated by monovalent interlayer cations and without water in the interlayer regions. Illite represents an interlayer‐deficient mica with reduced potassium content and structural formula close to K_0.6_Al_2_^[vi]^[]Si_3.4_^[iv]^Al_0.6_^[iv]^O_10_(OH)_2_, characterized by interlayer charges between 0.6 and 0.85 per formula unit [84, 87]. The non‐swelling illite structure contains both K^+^ ions (~80%) and some water molecules in interlayer positions [87].

Additionally, palygorskite and sepiolite are fibrous phyllosilicates with 2:1 *modulated* structures characterized by a continuous tetrahedral sheets with periodically inverted groups, creating discontinuous octahedral sheets and ribbon‐like polysomes, see Figure 2d [87]. This structural arrangement generates channels parallel to the fibre axis, resulting in high SSA [94]. Palygorskite, see Figure 2d, exhibits a dioctahedral character with ~20% octahedral vacancies, while sepiolite, see Figure 2e, shows a trioctahedral nature with almost complete octahedral occupancy [95, 96]. The ideal structural formulas are (Al_2_Mg_2_)Si_8_O_20_(OH)_2_(OH_2_)_4_·4H_2_O and Mg_8_Si_12_O_30_(OH)_4_(OH_2_)_4_·8H_2_O for palygorskite and sepiolite, respectively. Chemical composition varies significantly, with MgO contents ranging from 2 to 20 wt.% in palygorskites and 13–32 wt.% in sepiolites [95]. Moreover, these minerals form a continuous polysomatic series [95, 97].

**FIGURE 3:**
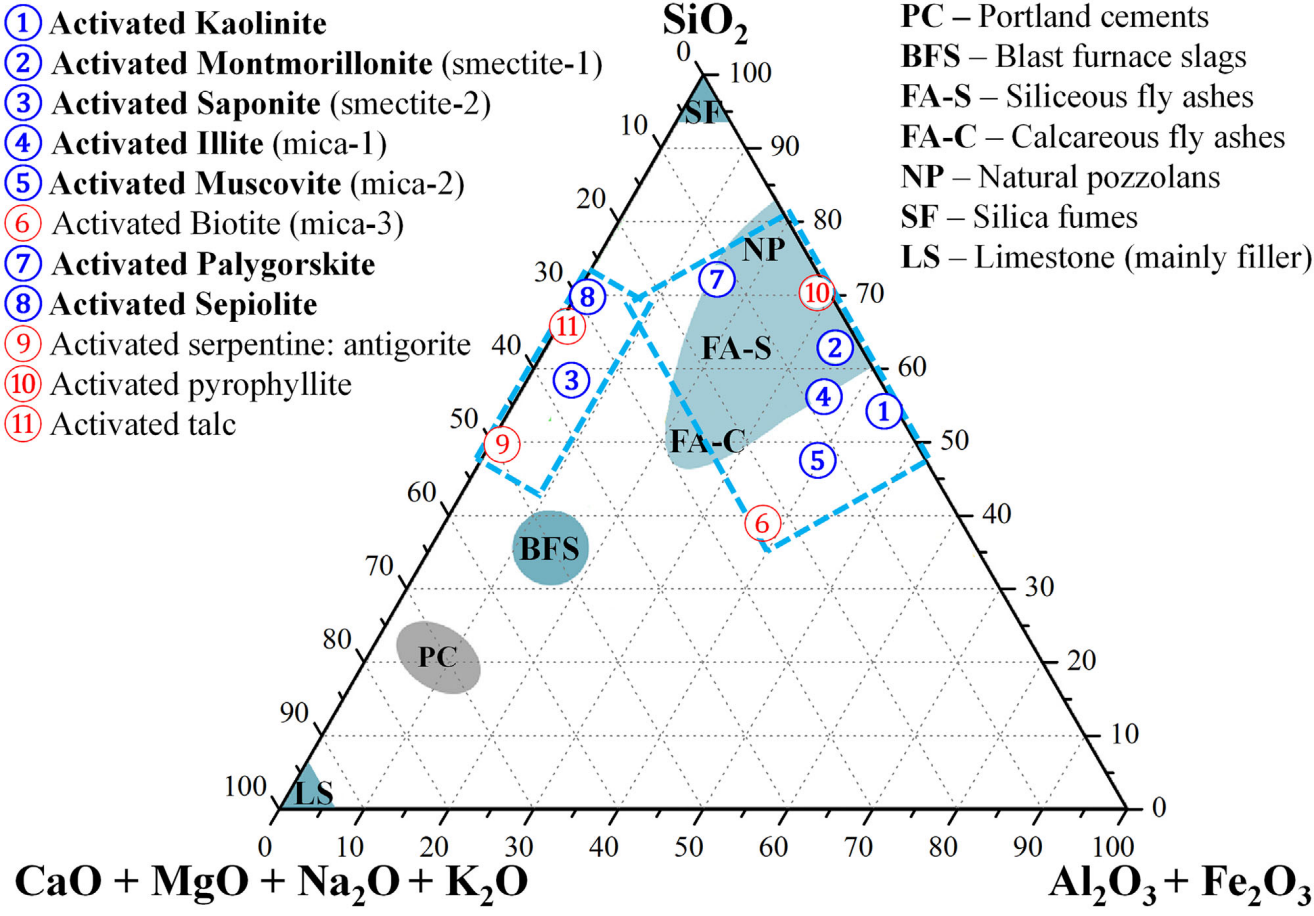
The chemical compositions for most typical activated clays, data from Table 5, in a ternary diagram (oxide percentages in wt.%). The positions in the diagram are given for “ideal” stoichiometries and slightly broader ranges are expected from the elemental substitutions. Clays highlighted in blue are those with proven pozzolanic activity in 2025. Clays highlighted in red are those where the pozzolanic activities are unknown to the authors of this work. Two clusters (Al‐rich and Mg‐rich) are highlighted. The positions for most common SCMs have been taken from ref. [10].

Raw clays constitute the basis for many SCMs but they require thermal, mechanical or chemical activations to achieve large pozzolanic activities (see sections 5 and 6). As will be further discussed in these sections, thermal activation, i.e., calcination, transforms raw clay minerals through dehydration, dehydroxylation, and amorphization processes that fundamentally alter their structures and chemical behaviours (for instance solubilities in basic media) [37]. Table 5 presents ideal chemical compositions of the main clay minerals after thermal activation. These compositions have been represented in a triangular diagram, see Figure 3, where the compositions of most used SCMs [10] are also displayed. The blue symbols in Figure 3 represent activated clay minerals with proven performances as SCMs.

**TABLE 5:** Ideal chemical formulas for selected thermally activated clay minerals. The main elemental compositions are given in oxide weight percentage and these values have been used to locate the different families in the phase diagram given in Figure 3.

Clay mineral	Ideal chemical formulas after thermal activation	SiO_2_ [wt.%]	Al_2_O_3_ [wt.%]	MgO [wt.%]	FeO [wt.%]	K_2_O [wt.%]	Na_2_O [wt.%]
Kaolinite ①	Al_2_Si_2_O_7_	54.1	45.9	—	—	—	—
Montmorillonite ②	Na_0.7_Al_4_Si_7.3_Al_0.7_O_22_	62.7	34.2	—	—	—	3.1
Saponite ③	Na_0.8_Mg_6_Si_7.2_Al_0.8_O_22_	58.5	5.4	32.7	—	—	3.4
Illite ④	K_0.6_Al_2_Si_3.4_Al_0.6_O_11_	56.0	36.3	—	—	7.7	—
Muscovite ⑤	KAl_2_Si_3_AlO_11_	47.4	40.2	—	—	12.4	—
Biotite ⑥	KMg_1.5_Fe_1.5_Si_3_AlO_11_	40.4	11.4	13.6	24.1	10.5	—
Palygorskite ⑦	Mg_2_Al_2_Si_8_O_21_	72.5	15.4	12.1	—	—	—
Sepiolite ⑧	Mg_8_Si_12_O_32_	69.4	—	30.6	—	—	—
Serpentine ⑨	Mg_3_Si_2_O_7_	49.9	—	50.1	—	—	—
Pyrophyllite ⑩	Al_2_Si_4_O_11_	70.2	29.8	—	—	—	—
Talc ⑪	Mg_3_Si_4_O_11_	66.5	—	33.5	—	—	—

The pozzolanic activities of kaolinites, smectites, micas, and palygorskites are firmly established. There is also one report on the pozzolanic features of sepiolite under chemical activation [98]. The red symbols in Figure 3 represent clay minerals which have so far not been reported to have pozzolanic activity. This could be due to unsuitable structures after treatments, limited abundance and/or lack of focused investigations. In any case and as illustrated in Figure 3, the compositional space for clay minerals with proven pozzolanic properties is very wide. Two large clusters can be identified: Al_2_O_3_‐rich minerals containing kaolinite, montmorillonite, illite, and muscovite; and (much less studied) MgO‐rich minerals comprising saponite and sepiolite.

The accurate determination of the amounts of clay minerals in complex natural samples is very challenging and space limitations prevent an in depth discussion here [76]. Here, one approach is briefly introduced. The amounts of crystalline components within raw clays can be determined using Rietveld quantitative phase analysis (RQPA) of X‐ray powder diffraction (XRPD) data [99]. The main limitations are: (i) the crystallinity degree of the different components; and (ii) the element substitution within the minerals (i.e., chemical variability). The overall amorphous fraction can be determined from the internal standard approach [100], typically employing α‐Al_2_O_3_ because of its similar X‐ray attenuation coefficient. However, more than one component can be nearly‐amorphous. The amount of (crystalline) kaolinite quantified through this approach should be interpreted as the minimum kaolinite content.

From the dehydroxylation mass loss, see below, a second clay mineral content estimation can be obtained. This should be interpreted as the maximum content as other minerals can contribute to the measure mass loss. Additionally, elemental analysis by X‐ray fluorescence (XRF) provides the total Al_2_O_3_ content, from which the Al_2_O_3_ attributed to other crystalline phases identified by RQPA, such as feldspars, plagioclase, and related aluminosilicates, can be subtracted. The remaining Al_2_O_3_ could then be attributed to total kaolinite (amorphous and diffracting). It is important to note that assumptions regarding the stoichiometry of these side phases may introduce bias; thus, the XRF‐derived estimate should be regarded as an upper bound. Taken together, the three estimations, from RQPA, TG, and XRF, allow for a more robust estimation of the kaolinite (or the clay mineral) content [47].

The initial stages of clay processing are fundamental in ensuring consistent quality and optimal performance of activated clays intended for their use as SCMs. Extraction typically involves open‐pit mining, with deliberate blending of stratified layers to achieve a homogenized raw material [76]. The handling of mine tailings and mineral waste is approached with a focus on minimizing environmental impact, prioritizing reuse or controlled disposal [101]. Upon delivery to the processing facility, the clay is further homogenized, commonly via blade milling or chevron stacking, to attain compositional uniformity. Pre‐drying and grinding operations are subsequently applied, tailored to the specific moisture content. The method of feeding the clay into the kiln (or the mill) is adjusted according to its plasticity and residual moisture, typically employing conveyors or bucket elevators. Collectively, these procedures constitute a broadly applicable processing sequence that precedes the activation step, exerting a direct influence on both the performance and sustainability of the resulting product [102]. The following two sections provide an overview of the thermal and mechanical activation protocols and results specific to selected clay types. More elaborate approaches, such as mechanothermal activation is not treated here [103].

## 5 Recent Progress in Thermal Activation (TA) of Clays

Thermal analysis, in its different modalities TG, DTA, DTG, is routinely employed for both the identification and the estimation of the amounts of clay minerals [104]. Thermal analysis curves may typically exhibit up to three distinct phenomena of interest: (i) dehydration (associated with mass loss and an endothermic signal) of loosely retained water and chemically bound water, (ii) dehydroxylation (also showing mass loss and endotherm(s)), and (iii) phase transitions, recrystallization and reactions between components (characterized just by thermal effects without any mass loss). For quantitative assessments, data should be normalized to dry mass, excluding the dehydration stage. Nonetheless, the initial step remains valuable for detecting secondary phases, such as 2:1 clay mineral in kaolinitic rocks. The TA signal is the composite answer to sample heating and therefore, the presence of several minerals results in effects that can be (are) overlapped. In any case, thermal analysis is useful for clay mineral estimation and also for helping to determine the optimum activation temperature window as this is located after (full) dehydroxylation but before any re‐crystallization/reaction that may inactivate the pozzolanic features by producing very stable phases.

**FIGURE 4:**
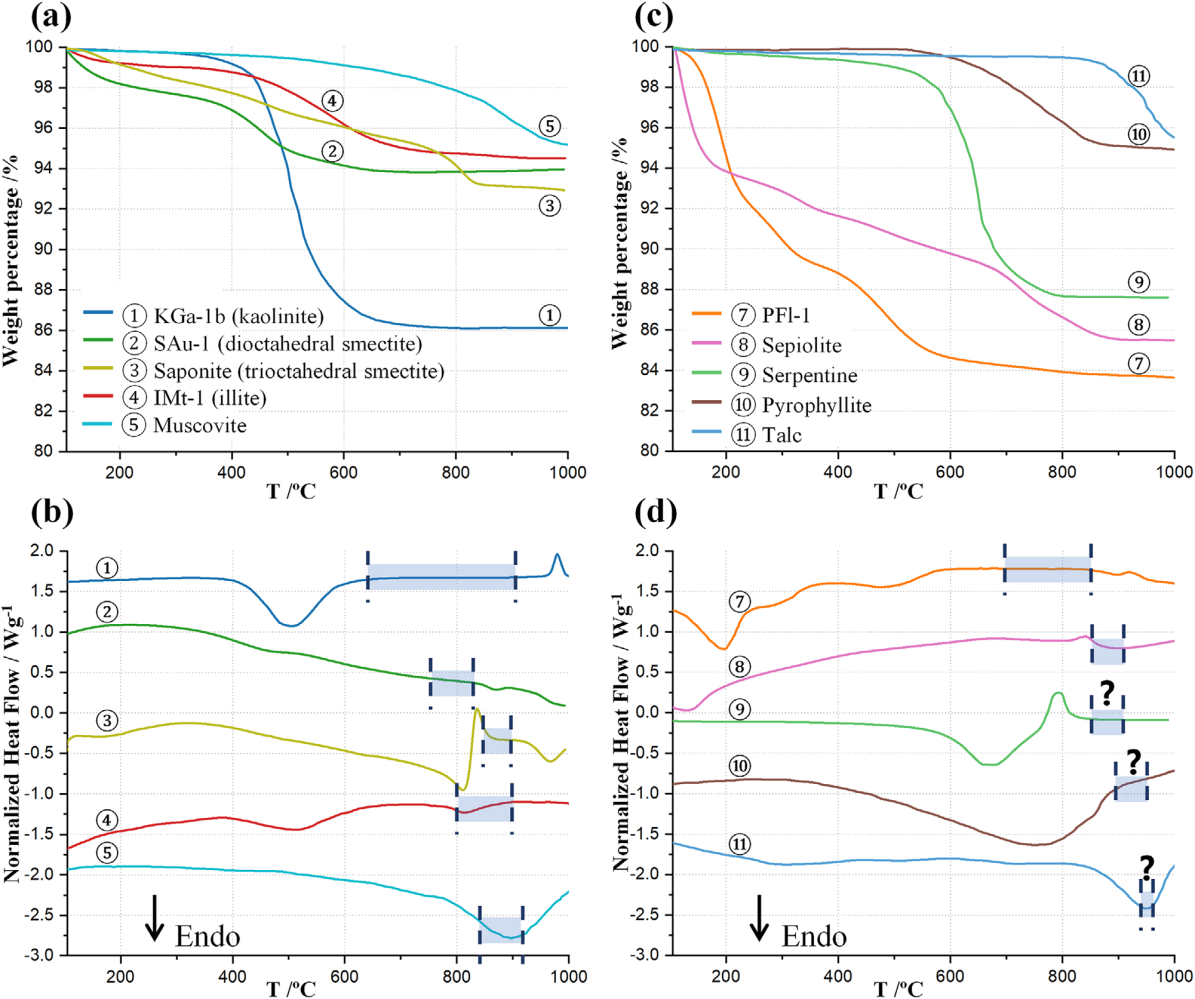
(a) and (c) TG and (b) and (d) DTA curves for selected clays. ① kaolinite KGa‐1b [105] from CMS; ② montmorillonite SAu‐1 [106] from CMS, ③ high‐grade saponite from ref. [47]; ④ illite IMt‐1 [107] from CMS, ⑤ high‐grade muscovite [108]; ⑦ palygorskite PFl‐1 [109] from CMS, ⑧ high‐grade sepiolite [110]; ⑨ high‐grade chrysotile [104] as a member of serpentine group; ⑩ high‐grade pyrophyllite [111] and ⑪ high‐grade talc [112]. The DTA curves in (b) and (d) have been vertically offset for clarity. Shaded regions in (b) and (d) indicate the commonly reported activation temperature windows for the corresponding clays (question marks highlight expected activation temperature windows, still not reported). Some data have been extracted from figures in the references using the Plotdigitizer application.

To illustrate this, Figure 4 displays the thermal analysis curves (thermogravimetry and heat flow) of selected high‐grade clay minerals including four standards of the CMS, all dried at 105°C. KGa‐1b, a low‐defect kaolinite clay standard, typically displays a dehydroxylation event within the 400°C–600°C range, see Figure 4a,b. Estimating kaolinite content from TG data requires several assumptions: (i) the absence of other minerals exhibiting mass changes within this temperature range, (ii) a defined stoichiometry for dehydroxylation, and (iii) low compositional variability in the raw material to ensure sample representativeness [76]. As an example, because the ideal chemical formula of kaolinite is Al_2_Si_2_O_5_(OH)_4_, the theoretical weight loss due to dehydroxylation is 13.9%. As expected from a high‐purity standard, KGa‐1b losses 14% of mass in the above‐reported temperature range, meaning 100% pure from TA. The situation is far more complex in commercial raw clays where several components/minerals may lose weight in the studied temperature range.

The primary objective of TA of clays is to maximize their pozzolanic reactivity. This is achieved targeting four critical sub‐goals: (i) dehydroxylation of the clay minerals; (ii) promoting partial or complete amorphization; (iii) avoiding recrystallization and/or reaction at higher temperatures, and (iv) preserving SSA as much as possible for enhancing early age reactivity. To satisfy conditions (i) and (ii), kaolinitic clays must be heated above the dehydroxylation threshold (∼650°C). However, the temperature must be below the recrystallization (iii) and extensive loss of SSA (iv), which are sample‐dependent, but usually close to 900°C. This TA temperature window is illustrated as a shaded region of ① line, see Figure 4b [105, 113].

For illustrative purposes, Figure 4 also displays data for two smectites, two micas and palygorskite, sepiolite, serpentine, pyrophyllite, and talc. As expected from their crystallochemistry, trioctahedral minerals invariably lose water at a higher temperature than their dioctahedral counterparts. The activation temperature windows, see below for detailed discussion, are also highlighted in Figure 4b,d.

**FIGURE 5:**
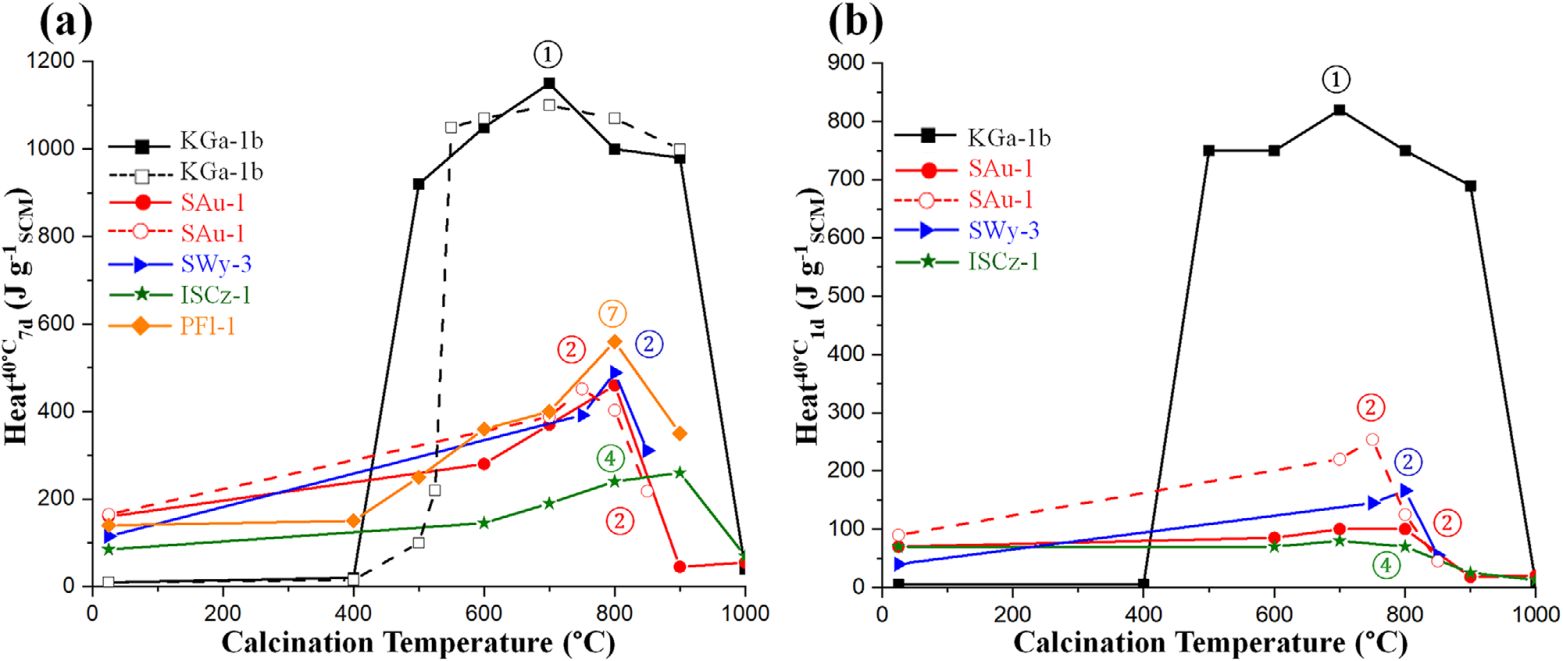
Cumulative heat release from the R^3^ test a) at 7 days and b) at 1 day as a function of calcination temperature for five standard clays from CMS. Some pristine clays display non‐negligible pozzolanic activity (i.e., without thermal treatment). Dashed lines and open symbols represent data from studies of the same material by independent research groups. Kaolinite KGa‐1b: solid line [113] and dashed line [109]; montmorillonite SAu‐1: solid line [113] and dashed line [106]; montmorillonite SWy‐3 [106]; interstratified illite–smectite ISCz‐1 [113]; and palygorskite PFl‐1 [109]. The optimum activation temperatures are 700°C, 750°C, 800°C, 800°C, and 800°C for KGa‐1b, SAu‐1, SWy‐3, ISCz‐1, and PFl‐1, respectively.

Next, the optimum TA temperature for having the highest pozzolanic activities is discussed. This can be inferred from the SAI values at 28 and/or 90 days, but this is a snapshot view. However, the R^3^ method provides this information in a shorter time, i.e., 7 days, and continuously with time for the cumulative heat. The R^3^ test, conducted at 40°C, is a standardized method [65] that has undergone extensive interlaboratory validation to establish its precision/accuracy features [48, 60, 64]. Thus, Figure 5 displays the R^3^ cumulative heats as a function of the calcination temperature for five clay mineral standards from CMS. The long‐term pozzolanic activity can be measured from the heat released at 7 days at 40°C. From Figure 5a, it can be deduced that the optimum calcination temperatures are 700°C, 750–800°C, 800, 900°C, and 800°C for KGa‐1b (kaolinite), SAu‐1 (montmorillonite), SWy‐3 (montmorillonite), ISCz‐1 (interstratified illite/smectite) and PFl‐1 (palygorskite). Moreover, for fast pozzolanic reactivity, the key performance indicator is the heat released in the R^3^ test at 12 h or 1 day [47]. Therefore, Figure 5b displays the cumulative heats at 1 day as a function of the calcination temperature. For interstratified illite/smectite, the small gain in heat from 800°C to 900°C at 7 days, it does not compensate the lower amount of heat released at 1 day. Therefore, it is concluded that 800°C is a better calcination temperature for this illite‐rich sample. Taking all data together, 800°C seems to be a temperature where pozzolanic activity is maximized for many clay minerals.

**FIGURE 6:**
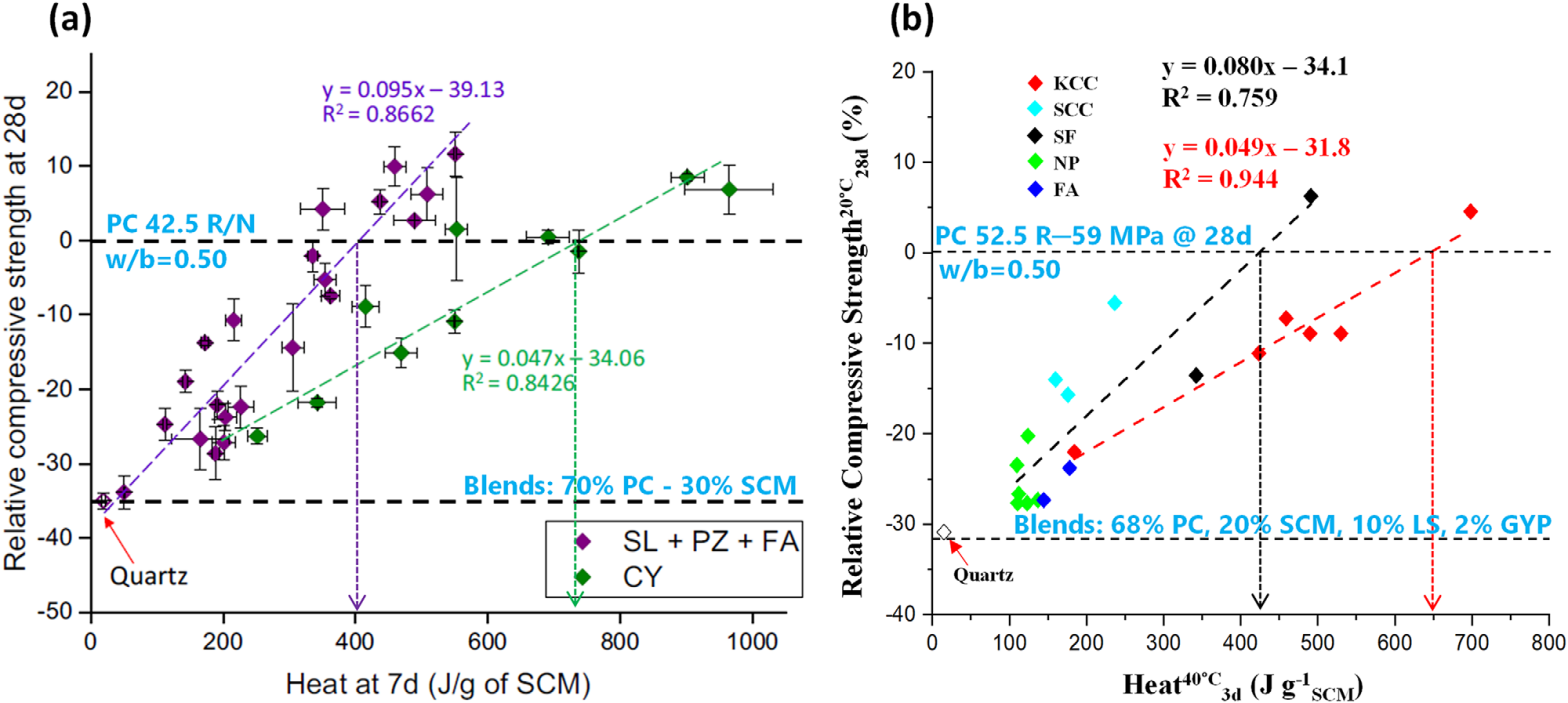
Correlation between the 28‐day Relative Compressive Strength (RCS at 20°C) and the cumulative heat release (R^3^ test at 40°C) for SCM‐containing blends. (a) Correlation between RCS at 28 days and R^3^ heat at 7 days for 70/30 blends, used under the terms of the Creative Commons CC BY license [60]. (b) Correlation between RCS at 28 days and R^3^ heat at 3 days for 68/20/10/2 blends, with permission from Elsevier B.V. [47]. Quartz used as inert reference in both studies.

Before discussing the thermal activation behaviour of selected types of clays, it is important to highlight findings from two independent studies that establish a correlation between the heat released in the R^3^ pozzolanic reactivity test and the compressive strength of blended cements [47, 60]. In both studies, approximately 30 wt.% of PC was replaced by SCMs, although the blend compositions and materials tested were different. Londono‐Zuluaga et al. (2022) employed a 70%PC‐30%SCM blends based on PC 42.5 R/N cements and evaluated kaolinitic calcined clays, natural pozzolans, and slags [60]. In contrast, Koufany et al. (2025) used a 68%PC‐20%SCM‐10%LS‐2%GYP formulation with a PC 52.5 R, incorporating both kaolinitic and smectitic calcined clays, along with natural pozzolans, fly ashes, and silica fume [47]. Despite these differences, both studies reported remarkably similar trends, as shown in Figure 6, where the heat released in the R^3^ test is plotted against the relative compressive strength (RCS). Notably, two distinct trends were observed in both of the studies: one associated with kaolinitic calcined clays and another with the remaining SCMs including smectitic calcined clays. For 70/30 blends (based on PC 42.5 R/N) and to achieve an RCS of 0%, kaolinitic calcined clays required approximately ~720 J g^−1^_SCM_ at 7 days, whereas the same value of RCS can be obtained for the other cluster (remaining SCMs) with ~400 J g^−1^_SCM_ at 7 days, see Figure 6a. Moreover, for 68/20/10/2 blends (based on a PC 52.5), RCS values of 0% are obtained for cumulative heat values at 3 days of 650 and 425 J g^−1^_SCM_, for kaolinitic calcined clays and the rest of SCMs, respectively, see Figure 6b.

Two important conclusions can be drawn: (i) the R^3^ cumulative heats of kaolinitic calcined clays develop less compressive strengths that the same value of SiO_2_‐rich SCMs. This is likely due to lower binding performances of carboaluminates when compared to C‐A‐S‐H gel. (ii) It is possible to predict a compressive strength at 28 days, based on the R^3^ heat developed by the SCMs (when fixing: (a) content of the SCM, (b) type of PC, and (c) w/b ratio), within a variability of ~10% for kaolinitic calcined clays and ~15% for the remaining SCMs. However, more systematic studies are needed to determine if other thermally‐activated clays (such as palygorskites or muscovites) are situated in these plots together with the calcined smectites.

With this knowledge and caveats in mind, Table 6 displays R^3^ heats (at different reaction times) for selected calcined clays of different families. As shown above, R^3^ heats are a good proxy for predicting compressive strengths of the blends but quantitative predictions for kaolinitic calcined clays cannot be directly translated to the other calcined clays. In any case and for similar active phase contents, with the knowledge that we have at the time of writing, the final pozzolanic activity of optimally calcined clays varies in the following sequence: kaolinites > palygorskites ~ Ca‐montmorillonite > Na‐montmorillonite > muscovites > illites > saponites. It is noted that mechanically activated muscovites, in optimal conditions see section 6.3, yield higher pozzolanic reactivities, comparable to the results for optimally calcined palygorskites.

**TABLE 6:** Pozzolanic activity of selected calcined clays determined by the R^3^ method and the modified Chapelle test, along with active phase content, calcination temperature, and key textural properties.

	Active phase [wt.%]	$Heat_{1d}^{40^{\circ }C}$ [J g^−1^_SCM_]	$Heat_{3d}^{40^{\circ }C}$ [J g^−1^_SCM_]	$Heat_{7d}^{40^{\circ }C}$ [J g^−1^_SCM_]	Chapelle test^a^	T_activation_ [°C]	D_v,50_ [µm]	SSA [m^2^ g^−1^]	Refs.
Kaolinite, KGa‐1b	96	n.r.	n.r.	≈1100	1400	800	n.r.	n.r.	[109]
High‐grade Kln	74	577	698	737	—	860	8.4	27.2	[47]
Medium‐grade Kln	40	302	490	539	—	860	12.0	4.0	[47]
Low‐grade Kln	20	98	184	225	—	860	12.0	4.5	[47]
Palygorskite, PFL‐1	79	n.r.	n.r.	∼530	950	800	n.r.	n.r.	[109]
Ca‐Montmorillonite	78	247	419	504	—	830	14.4	48.0	[52]
Na‐Montmorillonite	74	107	236	301	—	850	11.8	21.8	[114]
Saponite	80	90	160	191	—	850	11.8	51.9	[114]
Illite	67	150	201	237	—	840	15.8	71	[115]
Muscovite	57^b^	n.r.	n.r.	330	—	900	n.r.	8.4	[116]
Sepiolite	16^c^	n.r.	n.r.	n.r.	552^d^	800	n.r.	n.r.	[110]

*Notes*: n.r. stands for not reported.

^a^ Modified Chapelle test based on NF P18‐513 standard [110] – units mg Ca(OH)_2_/g of calcined clay.

^b^ Also contains illite.

^c^ Reactive silica determined by ASTM C379‐65 standard.

^d^ As references 1400 mg/g and 950 mg/g of calcined KGa‐1b and PFl‐1, respectively [109].

The thermal activation of kaolinites is very well documented and straightforward with optimum heating static temperatures of 750°C–800°C [102, 117, 118]. The calcinations are being carried out in rotary kilns or flash calciners but the discussion of the advantages and disadvantages of these experimental approaches are out of the scope of the present work [102, 119].

### 5.1 Smectite‐Based Clays

Research on smectite clays as SCMs has received much less attention than kaolinitic ones although their pozzolanic properties were well documented as early as 1996 [120]. In any case, recent studies have demonstrated that smectitic clays exhibit significant pozzolanic reactivity under appropriate activation conditions [106, 114, 121, 122, 123, 124]. However, as shown in Figure 4, the activation temperature range is narrower, i.e., 750°C–800°C for montmorillonites and 800–850 for saponites. It should be noted that the dehydroxylation temperature of smectites is governed by various factors, such as (i) particle sizes; (ii) structural disorder; (iii) the nature of the exchangeable interlayer cations: mainly Na^+^, Ca^2^^+^, etc. [106, 125, 126]; and (iv) last but not least the dioctahedral/trioctahedral character.

Raw smectites intercalates SP and this is highly detrimental for the rheological properties. Calcined smectites do not intercalate/absorb SP but they may still have high SSA, which is convenient for early‐age reactivity. However, SCMs in general, and calcined clays in particular, which present high SSA, pose significant challenges for the workability of mortars and concretes [127]. Mortars with calcined clays typically exhibit increased water demand and SP adsorption [128]. Thermal activation induces a reduction in SSA due to the structural collapse of the clay and the resulting smoother particle surfaces [129]. This effect is especially relevant for smectites, given their inherently higher SSA in the raw state [114, 130].

At this point, it is important to address whether calcined smectites intercalate SP or not. Some authors reported that SP is adsorbed only onto the edges of calcined 2:1 phyllosilicate (without intercalation/absorption) [131]. Moreover, the sorption of Ca^2+^ cations onto meta‐montmorillonite is less pronounced than in MK [128]. A very recent study has shown that smectitic calcined clays have SP requirements close, and in some cases lower ones, than those of calcined kaolinitic clays [114]. Therefore, there are no evidences of PCE intercalation in calcined smectites. This can be justified because the heating process beyond ≈700°C destroys the swelling behaviour although microstructures with layered features have been reported.

### 5.2 Mica‐Based Clays

On the one hand, the pozzolanic properties of calcined illite clays are well documented [124, 132, 133, 134]. Illite clays follow a similar thermal behaviour as described above for kaolinite and smectites, to yield meta‐illite below approximately 800°C–850°C [135], see Figure 4. However, when illite‐bearing clays also contain other clay minerals such as kaolinite, the optimum calcination temperature may slightly change [136].

On the other hand, there are fewer reports about the pozzolanic activity of TA muscovites [50, 137, 138, 139]. It is becoming very clear that muscovites activated mechanically outperform the same clays activated thermically. Therefore, muscovites will be further discussed when dealing with mechanical activation (MA).

Finally, and to the best of the authors knowledge, there are no reports dealing with the thermal activation of biotite clays.

### 5.3 Palygorskite‐Based Clays

The palygorskite group, the main mineral is known as attapulgite, is a hydrated magnesium aluminium silicate layered mineral, see Figures 1 and 2, with a complex crystal structure and fibrous morphology [140]. This phyllosilicate is notable for its discontinuous octahedral sheets which generate tunnels along the fiber axis, contributing to its high specific surface area, adsorption properties [94] and probably to its pozzolanic activity (after activation). The thermal data for palygorskite was given in Figure 4c,d, showing continuous weight loss up to 800°C due to physically adsorbed water, zeolitic water and the hydroxyl groups.

Several studies have shown that TA close to 800°C consistently yields optimum activation [109, 110, 141, 142, 143]. Palygorskites have relatively low Al_2_O_3_ content, see Table 5 and Figure 3, but quite high R^3^ heat values at 7 days, see Table 6. Two commercial samples containing 61 and 73% of palygorskite (and ~20% of smectite) gave R^3^_7d_ values of 500 and 550 J g^−1^_SCM_, respectively [144]. Not surprisingly, the 28d‐SAIs for the corresponding 80%PC‐20%SCM blends were quite high, 126 and 141%, but for a PC having just 39 MPa at that hydration time [144]. Research into the admixture requirements of this promising type of SCM is urgently needed.

### 5.4 Other Clays

Sepiolite, see Figures 1 and 2, is a hydrated magnesium silicate clay mineral with a distinctive fibrous morphology. Its crystal structure contains channels along the fiber axis, which host both zeolitic and coordinated water molecules. These channels contribute to sepiolite's high surface area and porosity and they are key for their adsorption and catalytic applications.

Figure 4 also displays the thermal behaviour of sepiolite which shows continuous weight loss up to 900°C [110]. However, two independent studies have shown that the pozzolanic activity at 800°C is slightly higher than that obtained by calcination at 900°C, likely due to the crystallization of enstatite at high temperatures [98, 110]. There are no R^3^ studies for activated sepiolites. However, it should be mentioned that sepiolite has also been chemically activated (acid treatment) [145].

To the best of the authors knowledge, there are no reports on the thermal activation of serpentines, pyrophyllites and talc to be used as SCMs.

## 6 Recent Progress in Mechanical Activation (MA) of Clays

Mechanical activation may offer a sustainable alternative to unlock the potential of unused clay deposits traditionally considered unsuitable (or challenging) for TA. This includes carbonate‐containing ones, muscovite and illite clays, or a mixture of clays where each of them shows a different optimum range of dehydroxylation/activation temperature as described in section 5. It seems that MA especially effective in the activation of 2:1 clays, such as muscovite and illite [134, 138] but it has also been used for kaolinite‐based clays [116, 146, 147] including low grade ones [148].

Mechanical activation could be a more sustainable method for activating clays than thermal processes, as less energy may be needed, particularly when electricity from renewable sources is utilized. For instance, the thermal decomposition of kaolinite needs an energy of ∼1600 kWh per ton of material vs. 200–1000 kWh for MA. However, these values are strongly dependent on the grinding medium (wet or dry) [149]; and more robust comparisons are needed.

It is a process that induces structural disorder, fracturing, and chemical modifications such as (partial) dehydroxylation through intensive grinding [36]. Unlike mechanical milling, which primarily reduces particle size (i.e., comminution) and creates dislocations or structural defects, MA achieves broader transformations, including amorphization [50]. The complexity of MA is reflected in various models that emphasize different mechanisms and influencing factors, illustrating its multifeatured nature [36]. There is no space here to discuss the mechanisms involved in MA.

Milling devices implement distinct stress modes (compression, shear, impact, and collision) to transfer as much energy as possible to the material. Both the initiation and evolution of the MA process are highly dependent on the type of mill and milling conditions (milling rate and time, ball/powder weight ratio, filling ratio, etc.). Different mills have been used for MA [150], such as the planetary ball mill [146, 150, 151, 152, 153], disc/oscillating mill [148], traditional ball mill [147], “Los Angeles” ball mill [154] and ultra‐centrifugal mill [155], where the former seems to be the most energy‐efficient equipment. A recent work demonstrated the successful use of an attrition mill in the MA of illite [134]. The grinding conditions and the type of clay affect the efficiency of each mill [156]. Dry grinding resulted more effective than a wet medium in the amorphization of kaolinite using a planetary ball mill [150]; but the effectiveness increases by increasing the water/powder ratio, needing longer milling times, and also by increasing the ball‐to‐powder mass ratio (BPR) [150]. Both the efficiency of the mill and the industrial upscaling feasibility are key parameters, where tumbling ball mills, although they work at lower speeds than planetary ball mills, allow larger amounts of materials [157], and also the attrition mill [134] could be more easily scalable. The total energy transferred to a material (i.e., clay) is a very interesting topic but not within the scope of this work.

By optimizing MA parameters ‐grinding experimental conditions including the use of grinding aids‐ it is possible to achieve comparable or superior pozzolanic reactivity to that of thermally activated materials for some types of clays. However, there is a much smaller body of research on MA than on TA, and especially focused on the use of grinding aids in MA, such as TEA [147]. Grinding aids have the potential to improve grinding efficiency by reducing particle agglomeration and improving contact points. However, a comprehensive understanding of the admixture effects on grinding performances is lacking. The agglomeration during strong milling also comes with fracture [151]. Some studies have shown that MA significantly enhances the early‐age reactivity of kaolinite, smectite, and muscovite clays compared to thermal activation. This improvement may be primarily attributed to the increased external surface area, which markedly accelerates dissolution rates which enhances early‐age pozzolanic reactivity [36, 52, 151]. For instance, mechanically activated clays consume portlandite more rapidly, particularly during the first hours to days of reaction, underscoring their enhanced pozzolanic activity [146].

TA has a low impact on the morphology of clays. However, during MA, the measured particle size decreases and the SSA increases up to a point, and after that, they are kept constant or may even decrease. Additionally, the particle morphology is modified with the MA, making them more spherical [36, 52, 146]. This change results from high‐energy impacts during milling, which promote reshaping and particle agglomeration. Interestingly, and perhaps counterintuitively, it seems that the amount of required SP is not much larger for processing the corresponding binders. When comparing MA and TA kaolinitic clays, less amount of PCE‐SP was required for the *pastes* based on MA kaolinitic clays [146]. However, the same authors have reported slightly larger requirements of PCE‐SP for a muscovite‐based MA sample, when compared to the same clay but processed by TA [139]. More comparative studies are needed and with a focus on the corresponding *mortars*.

Baki et al. [50] demonstrated that MA is particularly effective in enhancing the pozzolanic activity of 2:1 clays, such as smectite and muscovite. This improvement is attributed to the structural changes induced by MA, including increased amorphization and the creation of reactive sites, which significantly boost their reactivity in cementitious systems. Unlike thermal treatment, MA could achieve nearly complete amorphization of these clays [50]. MA can produce (partial) dehydroxylation of clay minerals, where hydroxyl groups are lost and the released water could be partly re‐adsorbed by the clay; consequently, the dehydroxylation peak of the phases will be less pronounced and shifted to lower temperatures [134, 153, 158]. The combination of thermal and mechanical activations have been proposed. However, comparisons should be done with care as for example in ref. [159] two heterogenous clays were treated by TA, MA, and MA followed by TA. The two TA clays had D_v,50_ values bigger than 50 µm, and therefore they obviously did not show significant pozzolanic activity (in spite of a kaolinite content of ~20 wt.%). Comparisons should be carried out for TA clays with particle sizes close to that of the cement, i.e., 10–15 µm. There are many reports dealing with TA clays with D_v,50_ values bigger than 20 µm, which unsurprisingly show (very) low pozzolanic activities. The effects of MA on kaolinite‐, smectite‐, muscovite‐, illite‐ based clays are illustrated in Table 7. This table shows the pozzolanic activity of selected MA clays as determined by the R^3^ method, including additional relevant information.

**TABLE 7:** Pozzolanic activity of selected mechanically activated clays determined by the R^3^ method, along with information about the active phase/s, and textural properties. ACn stands for amorphous and crystalline not‐determined overall content.

	Active phase [wt.%]	ACn [wt.%]	Fully amorphous	$Heat_{1d}^{40^{\circ }C}$ [J g^−1^_SCM_]^a^	$Heat_{7d}^{40^{\circ }C}$ [J g^−1^_SCM_]^a^	Milling details	D_v,50_ [µm]^a^	SSA [m^2^ g^−1^]^a^	Refs.
High‐grade Kln	82	93	Yes	536	628	a)	2.7	10.0	[146]
High‐grade Kln	71	66	No	509	605	b)	25^b^	23.0	[147]
High‐grade Kln	73	54	No	250	485	b)	30^b^	40.0	[147]
High‐grade Kln	74	n.r.	Yes	600	680	c)	3.0	7.4	[151]
Medium‐grade Kln	63	n.r.	Yes	225	480	c)	0.1^c^	10.6	[151]
Ca‐Mont	78	n.r.	Yes	312	403	c)	3.8	20	[52]
Na‐Mont	61	n.r.	Yes	297	394	c)	3.7	14	[52]
Muscovite/illite	57	91	Yes	445	561	d)	4.5	9.0	[138, 139]
Illite	n.r.	n.r.	Partial	218	254	e)	9.0	n.r.	[134]

*Notes*: a) Planetary ball mill (PM 400, Retsch), zirconia jars (125 mL), 350 rpm/120 min, BPR = 20:1, 10 mm zirconia balls, 20 vol% occupancy (balls + powder); b) Ball mill, 60 rpm/12h. 0.15 wt.% TEA. Three‐stage process: i) BPR = 17:1 (0–4 h), ii) BPR = 22:1 (4–8 h) and iii) BPR = 33:1 (8–12 h). Total grinding time: 12 h; c) Planetary ball mill (PM 100, Retsch), stainless steel jar (500 mL), 500 rpm/1 h, BPR 25:1, 2 mm stainless steel balls; d) Planetary ball mill (PM 100, Retsch), zirconia jar (500 mL), 400 rpm/90 min, BPR = 20:1, 10 mm zirconia balls, 20 vol% occupancy (balls + powder); e) 6L‐attrition mill, two‐stage milling (BPR = 20:1): i) 350 rpm/30min, 20 mm ball diameter, ii) 500 rpm/30min, 10 mm balls. n.r. stands for not reported.

^a^ Some data have been extracted from figures in the references using Plotdigitizer application.

^b^ Agglomerated.

^c^ Very low but given in the publication.

### 6.1 Kaolinite‐Based Clays

Kaolinite clays after MA are quite different to the corresponding TA clays (physically, chemically, and structurally), not only in particle size/SSA, morphology, and water content, but also in the coordination environment of aluminium. For instance, in MA kaolinitic clays, the Al mainly remains in octahedral coordination [152]. TA kaolinitic clays exhibit high reactivity due to complete dehydroxylation, which creates an amorphous phase, i.e., metakaolin [152], see section 5. The MA process also strongly affects the dehydroxylation process of kaolinite. The dehydroxylation peak, as seen in the DTG trace, is wider, less symmetric and it appears at lower temperatures [158]. The amorphization degree of kaolinite‐based clays can be monitored by the full width at half maximum (FWHM) of the (001) reflection, which corresponds to the main kaolinite diffraction peak (located at 12.3°/2θ using Cu_Kα_ radiation) [147, 160]. The kaolinite amorphization degree (KAD) can be quantified by powder diffraction when comparing the results before and after MA. Interestingly, Muzenda et al. [147] found that mortars, prepared with MA kaolinitic clays with KAD = 74%, showed similar compressive strengths than the mortars prepared with the TA clay (fully amorphized). When the KAD value of the MA‐clay was 58%, the compressive strength corresponded to 90% of the TA‐clay mortar at 28 days, but similar at 90 days. MA could be a better activation approach for raw clays with well‐ordered kaolinitic structure [147].

Mechanically activated kaolinites show competitive pozzolanic features (as measured by the R^3^ tests) when compared to the TA results [146, 147, 151], see Table 7. This is possibly due to the higher SSA of the MA kaolin clays which accelerates the pozzolanic reaction rates [152]. In binary PC‐SCMs blends (replacement rates of 20 and 30 wt.%), MA‐mortars showed lower strengths than the corresponding TA‐mortars [161]. In summary, and to the best of the authors knowledge, there are several reports showing the MA of kaolinitic clays but they have not outperformed their TA counterparts.

### 6.2 Smectite‐Based Clays

There are several reports on the MA of smectites [50, 52, 162] which seems to produce SCMs with slightly enhanced pozzolanic activities when compared to the results yielded by the corresponding thermally‐activated clays. The R^3^ test showed that Ca‐ and Na‐montmorillonites can effectively produce SCMs by MA [52], see Table 7. Na‐ and Ca‐montmorillonite clays, after MA, showed similar heat curves and they yielded much higher heat values at 1 day than the corresponding TA montmorillonites. However, at 7 days, the thermally‐activated Ca‐montmorillonite developed slightly higher heat [52]. Based on the published papers, which are not many, it can be stated that for smectites, MA can yield at least as competitive SCMs as TA. They could be even better as the pozzolanic reaction kinetics is faster. However, the SP requirements for MA smectites are unknown. Research on the features of mechanically‐activated smectitic LC^3^‐50 mortars (PCE‐SP contents for similar flow values and compressive strengths) is urgently needed.

### 6.3 Muscovite‐Based Clays

Muscovite clays are reported to benefit much more from MA than from TA, achieving greater structural disruption and reactive site generation [50, 138, 139]. For example, the R^3^ test indicates that the MA of (kaolinite‐containing) muscovite clay gave ~560 J g^−1^_SCM_ at 7 days, meanwhile the same clay activated at 900°C yielded ~325 J g^−1^_SCM_ [138]. LC^3^‐50 pastes incorporating MA muscovite surpassed those with TA muscovite in compressive strength at 3 and 28 days. The compressive strengths at 3 days were 42 and 22 MPa for blends containing MA and TA clays, respectively. At 28 days, the corresponding values were 54 and 41 MPa, respectively [139]. Similar results were obtained by Baki et al. [50] for mortars based on 80%PC and 20%SCMs blends. All published works so far indicate that MA is more effective than TA for muscovite clays. We speculate that the very high temperature required for complete dehydroxylation, close or above 900°C see Figure 4, already starts to develop deactivation. This advantage of MA over TA could be tested with talc, which also requires temperatures above 900°C for effective dehydroxylation, see Figure 4. However, we acknowledge that talc has no potassium, which can be very important for activation.

### 6.4 Illite‐Based and Other Clays

The MA processing of illite [163] induced significant structural alterations as grinding time increased (including decreased surface area due to smoothing of the surface and agglomeration). Comparing MA of an illite‐rich clay (~67 wt.% of illite) with TA [134], higher cumulative heat R^3^ values are obtained by optimized MA (220 and 250 J g^−1^_SCM_ at 1d and 7d, respectively) than for TA (65 and 90 J g^−1^_SCM_ at 1 and 7 days, respectively for activation at 800°C for 2 h, and 30 and 110 J g^−1^_SCM_ for the activation at 900°C for 2h). As in the case of kaolinite, the degree of amorphization depends on the milling conditions, and the dehydroxylation peak of illite (530°C) appeared at lower temperature and less intense by increasing the milling [134]. Water demand of mortars prepared with 30 wt.% of MA clay replacement (w/b = 0.485 and sand‐to‐binder mass ratio of 2.75) was slightly lower than that for mortars based on 30 wt.% of TA clay replacement. Finally, mortars prepared with different replacement percentages (0–40 wt.%) of optimized MA‐illite clay show at 28 days, an increase of compressive strengths from 40 MPa (no replacement) to 45 MPa for 40% replacement. However, when the PC is replaced by the TA‐illite clay (900°C/2 h), the compressive strength decreases to 27 MPa (for 40% replacement) [134]. In terms of both R^3^ values and compressive strengths, and for the studied illite‐rich clay, MA outperformed TA.

To the best of the authors knowledge, there are no reports on the MA of palygorskites, sepiolites, serpentines, pyrophyllites and talc to be used as SCMs.

## 7 General Correlations for Predicting the Compressive Strength of Mortars

Section 1 introduced the need of low‐carbon cements, a key approach being a large replacement ratio of Portland clinker by SCMs with pozzolanic activity (measured as detailed in Section 3). The raw clays, described in Section 4, have little, if any, pozzolanic behaviour. Hence, clays require activation, which in this context means to transform them in SCMs: (i) with the largest possible pozzolanic activity; (ii) with the minimum cost; and (iii) in the most sustainable manner. Sections 5 and 6 have shown the recent advances in thermal and mechanical activations of clays. To measure the pozzolanic reactivity and kinetics, R^3^ measurements are emerging as the key assessment method. However, the ultimate goal is to predict the mechanical strengths at given hydration times (and other performances in the future) by knowing the components of the blends and taking into account the key experimental variables: (a) characteristics of employed PC, (b) characteristics of the SCM, (c) PC/SCM/LS ratios; (d) sulfate balance; (e) w/b ratio; and (f) temperature; just to mention the most important ones.

Section 5 already detailed a correlation between the relative compressive strengths at 28 days and at 20°C and the cumulative heats obtained from R^3^ assay at 40°C, for a Portland clinker replacement of ~30 wt.% and w/b = 0.50. It was shown that the results cluster in two groups, one for kaolinitic calcined clays and the other for the remaining SCMs including smectitic calcined clays. This showed that R^3^ values can be good descriptors to predict mechanical strengths. However, there was a knowledge gap on this type of correlation for the most important LC^3^ cements, those with clinker replacement close to ~50%.

Therefore, in this section, we built on the previous findings by compiling reported data for LC^3^‐50 (i.e., PC replacement of ~50 wt.%). This allows us to discuss two key features: the role of fineness of the PC, PC42.5 vs. PC 52.5; and the role of the w/b ratio. The ultimate goal is to build knowledge to predict mechanical strengths of commercially‐available low‐carbon cements.

### 7.1 General Correlation of Compressive Strengths and Pozzolanic Performances for LC^3^‐50 Binder with w/b = 0.50

**FIGURE 7:**
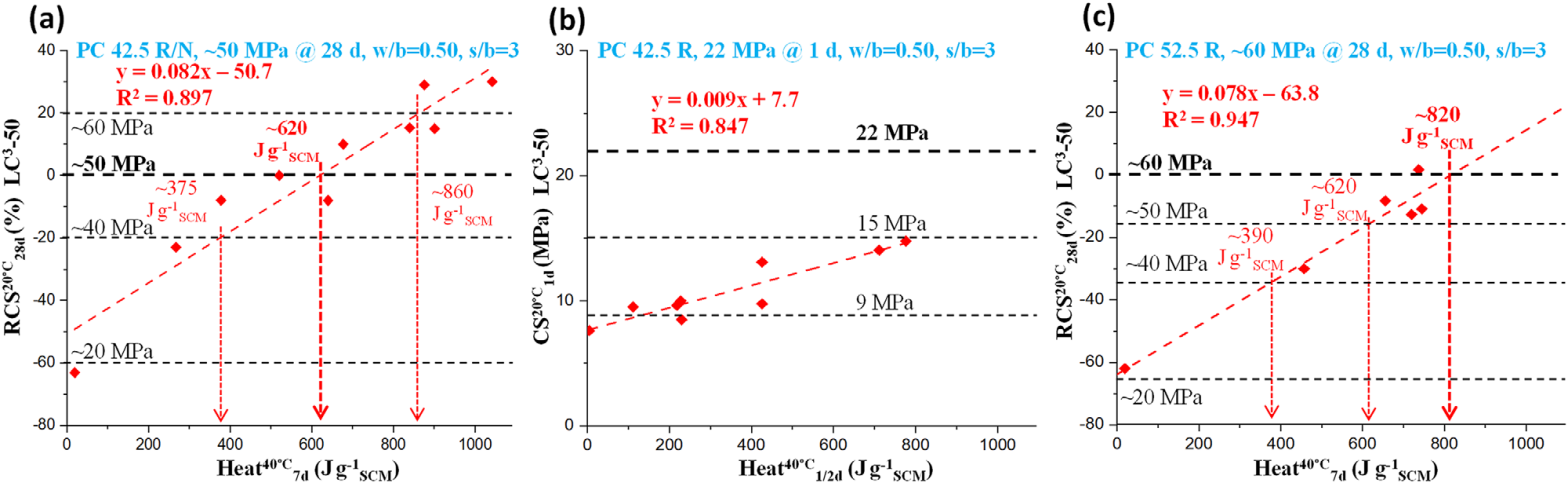
Correlations between mechanical strengths at 20°C and the cumulative heat release ‐R^3^ test‐ at 40°C for LC^3^‐50 blends based on kaolinitic calcined clays. All data for mortars with w/b = 0.50 and s/b = 3.0. a) Correlation of Relative Compressive Strengths at 28 days of low‐carbon cements based on PC 42.5 vs. Heats at 7 days, data from refs. [63, 164, 165]. b) Correlation of the Compressive Strengths at 1 day of low‐carbon cements based on PC 42.5 vs Heats at 12 h, data from refs. [63, 166]. c) Correlation of RCS at 28 days of low‐carbon cements based on PC 52.5 vs. Heats at 7 days, data from refs. [147, 167]. The data for the blend with quartz, i.e., without pozzolanic reactivity, in panels a) and c) have been specifically obtained for this work.

The gradual increase in compressive strength with increasing calcined kaolinite content, and so with the heat released as measured by the R^3^ assay, is well known since 2016 [63]. It is also known that the LC^3^‐50 binders based on PC 42.5 R/N yielded similar compressive strengths (or higher) when using clays with 50% of kaolinite content (or higher) [13]. Here, we build on these findings. Figure 7a includes Avet et al. seminal work data [63], recent contributions [164, 165] and new results from our laboratory obtained using an inert dilution, i.e., quartz with 19 J g^−1^_SCM_, with a PC 42.5 R with 50.2 MPa at 28 days. A linear trend is observed and calcined clays yielding ~620 J g^−1^_SCM_ result in RCS = 0. Knowing that MK releases about 1100 J g^−1^_SCM_, see Figure 5a, it is straightforward to conclude that clays with kaolinite contents of ~55 wt.% (or higher) will develop RCS values of 0 (or higher).

Figure 7b displays the correlation of the compressive strengths at 1 day with the R^3^ heat released by the activated clay at 12 h. It can be seen that for most commonly used kaolinitic clays, the resulting compressive strengths are quite low, ranging 9–15 MPa. In fact, it has been identified that the low compressive strengths at 1 day of hydration, are a key drawback for LC^3^‐50 binders [10]. This could be mitigated by using PC 52.5 to fabricate LC^3^‐50 binders, but there are far fewer reports using this type of PC. This could be surprising as using a more reactive PC seems to be a natural way to improve early age strengths. However, PC 52.5 could be less suitable to fabricate LC^3^‐50 binders.

Figure 7c displays the RCS values for PC 52.5‐based LC^3^‐50 binders where the R^3^ heats are also reported (for mortars with w/b = 0.50). As expected, the quality/grade of the raw clay has to be higher for attaining mortars with RCS = 0, i.e., 60 MPa. This plot indicates that a kaolinitic calcined clay releasing ~820 J g^−1^_SCM_ is needed to produce such a mortar. This translates to an effective kaolinite content of ~75 w%. However, it is also worth highlighting the differences in the cuts with the *y*‐axis. On the one hand, for PC 42.5, the ordinate in the origin is −50.7%. This means that an inert dilution, without pozzolanic effect, should result in a compressive strength of ~25 MPa. On the other hand, for PC 52.5, the ordinate in the origin is −64%. This means that an inert dilution should result in ~22 MPa. These results are unexpected, given that PC 52.5 is more reactive than PC 42.5. Therefore, further investigations are needed to clarify the origin of this behaviour, that could be due to unavoidable variability when compiling data from different cements. Our results give 23.1 and 18.5 MPa, for the quartz‐containing blends using PC 52.5 and 42.5 cements, respectively. Moreover, for PC 52.5‐based LC^3^‐50 binders to attain ~50 MPa (i.e., RCS of −16%), a kaolinitic calcined clay releasing ~620 J g^−1^_SCM_ is needed. This strength is obtained for PC 42.5‐based LC^3^‐50 binders with a kaolinitic calcined clay releasing ~620 J g^−1^_SCM_. According to these plots, in order to attain ~40 MPa, the heats released by the kaolinitic calcined clays should be ~375 and ~390 J g^−1^_SCM_ for LC^3^‐50 binders based on PC 42.5 and PC 52.5, respectively. More robust research is needed to add data to firmly establish this intriguing behaviour. We have an ongoing investigation focused on understanding the possible different behaviour of PC 42.5 and PC 52.5 in LC^3^‐50 binders.

### 7.2 General Correlation of Compressive Strengths and Pozzolanic Performances for LC^3^‐50 Binder with w/b = 0.40

In many cases, the water‐to‐binder ratio used in field applications is smaller than 0.50. Adding less water has the advantage of resulting in smaller (residual) porosities. In turn, less porosity yields higher compressive strengths and generally better durability performances. There is no space here to discuss the durability performances of low‐carbon cements based on activated clays. Thus, in this section, we discuss the correlations of compressive strengths (which is also a proxy for durability performances) with the pozzolanic activity of calcined clays for mortars with w/b = 0.40.

**FIGURE 8:**
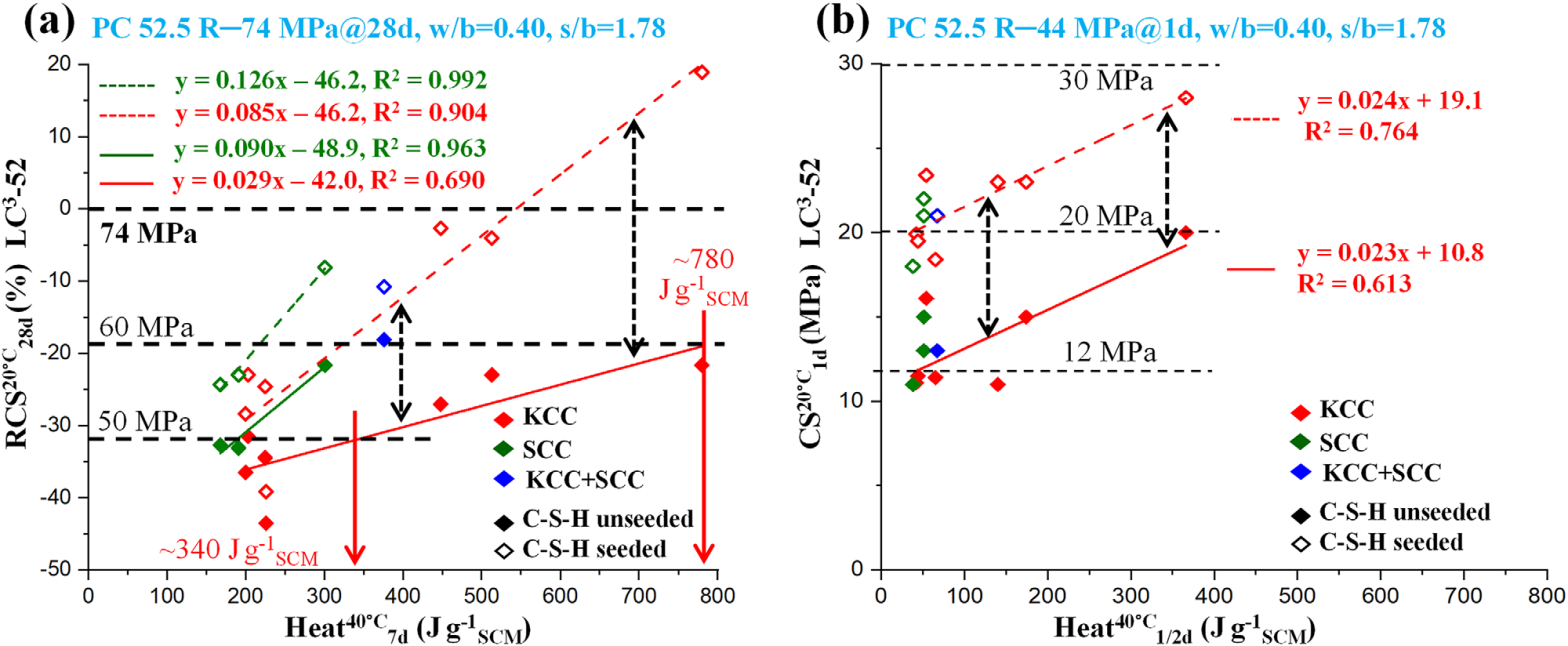
(a) Correlation plot for the relative compressive strengths (%) measured at 28 days at 20°C with the R^3^ cumulative heats obtained at 40°C and 7 days. Qz (in black) corresponds to the reference mix (without pozzolanic activity) and is included in the fitting of both KCC and SCC series. (b) Correlation plot for the compressive strengths (MPa) measured at 1 day at 20°C with the R^3^ cumulative heats obtained at 40°C and 12 h. All data for mortars with w/b = 0.40 and s/b = 1.78 compiled from the following works carried out at Universidad de Malaga [114, 167, 168, 169, 173]. KCC stands for kaolinitic calcined clay (red symbols). SCC stands for smectitic calcined clays (green symbols). The open symbols correspond to data obtained by adding 2%, by the weight of binder, of Master X‐Seed STE53 (a technology based on C‐S‐H nucleation seeding). The blue symbols correspond to a clay which is a mixture of kaolinite and smectite.

Figure 8a plots data of RCS‐28d (at 20°C) as a function of the R^3^‐heat‐7d (at 40°C) from all the work carried out at Universidad de Malaga with the following experimental conditions: LC^3^‐52, PC 52.5‐based, mortars with standard sand, w/b = 0.40 and s/b = 1.78 [114, 167, 168, 169]. The compressive strengths of the same samples after adding a strength‐enhancing admixture (2% by the weight of binder of Master X‐Seed STE53) are also plotted. Three main conclusions can be drawn from these data: (a) As shown in Figure 6, the behaviour of kaolinitic calcined clays is different from that of smectitic calcined clays. For attaining a target RCS, the kaolinitic calcined clays need to develop significantly higher R^3^‐heat. (b) C‐S‐H nucleation seeding enhances the mechanical strengths, not only at early ages (see below) but also at 28 days. Moreover, the slopes of the C‐S‐H seeded mortars are significantly higher than those of the unseeded one. We interpret this result as the seeding is also enhancing/activating the pozzolanic reaction and not only the PC hydration. However, more research is needed to mechanistically understand this behaviour. We speculate that a more homogeneous portlandite distribution in the seeded mortars may lead to a more homogeneous pozzolanic reaction in the samples enhancing the mechanical properties. We are carrying out a research program using X‐ray micro‐ and nano‐tomography (in pastes!) to try to understand this behaviour [170, 171, 172]. (c) When comparing the data of Figure 8a with those plotted in Figure 7c, it is clear that smaller w/b ratios (i.e., 0.40) increase the compressive strengths, but not dramatically. For instance, to attain 60 MPa, the required heats for the kaolinitic calcined clays are ~780 and ~820 J g^−1^_SCM_ for LC^3^‐50 binders fabricated with w/b ratios of 0.40 and 0.50, respectively. The difference is larger for a 50 MPa target, where the required heats are ~340 and ~610 J g^−1^_SCM_ for binders made with w/b ratios of 0.40 and 0.50, respectively. Noteworthy and for LC^3^‐50 binders based on smectitic calcined clays, to attain a target of 50 MPa with w/b = 0.40, a calcined clay with the very modest value of ~200 J g^−1^_SCM_ will be fine. This is 41% lower R^3^ heat than the corresponding kaolinitic calcined clay.

Our studies of LC^3^‐52 binders also include compressive strength data at 1 day, in the same experimental conditions, for seeded and unseeded mortars. All published data [114, 167, 168, 169] are plotted in Figure 8b. We decided to plot the 1d‐CS, measured at 20°C, versus the R^3^‐heats at 12 h, measured at 40°C, because the correlation is a bit better. It is noted that we have not excluded any data, even a clay which is a mixture of kaolinitic and smectitic minerals is displayed, although their numerical values have not been used in the fits. Several conclusions can be drawn from these data: (a) For kaolinitic calcined clays, the compressive strengths are increased due to the pozzolanic reaction but only for materials releasing ~150 J g^−1^_SCM_ (or more) at 12 h in the R^3^ test. (b) There is need for more data, smectitic calcined clays releasing more than ~100 J g^−1^_SCM_ at 12 h, to firmly establish if the different behaviour between kaolinitic and smectitic calcined clays is also evident in the RCS at 1 day. (c) C‐S‐H nucleation seeding enhances the early mechanical strength development due to the acceleration of PC hydration. However, the similar slopes of the strength evolution lines for seeded and unseeded mortars suggest that the pozzolanic reaction is not significantly promoted at 1 day of hydration. This observation is consistent with the inherently slower kinetics of the pozzolanic reaction. (d) We cannot infer if C‐S‐H nucleation seeding is more effective at w/b = 0.40 or w/b = 0.50, due to the lack of robust published works for this last water‐binder ratio. More comprehensive research is needed to enhance the LC^3^‐50 hydration with different water/binder ratios and with different admixtures (i.e., C‐S‐H nucleation seeding, alkanolamines, etc.).

## 8 Challenges in Optimizing Low‐Carbon Cements Based on Activated Clays

Low‐carbon cements based on thermally activated clays, the so‐called LC^3^‐50 binders, are becoming accepted and commercialized [119]. However, there are several challenges that must be overcome to achieve widespread acceptance in the risk‐averse, highly competitive, globally arranged, and standardized but locally‐based, construction field. Here, we highlight some of them. First, as is widely reported, the compressive strengths of mortars [63] and concretes [117, 174] at 28 days, for calcined clays with enough reactivity, are similar to those of neat PCs or higher [13, 35, 63]. However, the early age strengths of LC^3^‐50 mortars are very low, see Figures 7b and 8b. From a cement producer's viewpoint, the early strengths are measured at 2 days. From a concrete manufacturing viewpoint, the early strengths are generally measured at 1 day. Here, we focus on the concrete approach, which is the final product. Hence and for neat PCs, the compressive strengths of mortars range from ∼10 MPa (for PCs 42.5 N, w/b = 0.50) [175] and ∼20 MPa (for PCs 42.5 R, w/b = 0.50) [63] to 35–45 MPa (for PCs 52.5 R, w/b = 0.40) [168]. For LC^3^‐50 binders, the reported strengths of the mortars at 1 day are usually less than half, ranging from 10–15 MPa (for PC 42.5 R and w/b = 0.40) [166] to 11–20 MPa (for PC 52.5 and w/b = 0.40) [168]. For that reason, strength‐enhancing admixtures, such as C‐S‐H nucleation seeding [176, 177, 178, 179] or alkanolamines [180, 181], are being used. Although there have been encouraging results [114, 168, 169, 182], more research is required to cost‐effectively increase the 1‐day mechanical strengths for LC^3^‐50 mortars and concretes. Other approaches, like steam curing [183], are possible but due to space limitations the advantages and drawbacks are not discussed here. Second, it is also known that, in many cases, LC^3^‐50 binders, based on kaolinitic calcined clays, suffer a slump retention loss [17]. This is the decrease in workability (flow) of concrete during the first 60–120 min. This effect can be corrected with state‐of‐the‐art PCE‐SPs [114, 168, 169, 184] but more research is needed to prove the general applicability of this approach and to gain a larger portfolio of tailored SPs. However, the cost implications of employing new SPs are not negligible, see Section 1, which may limit their use. Moreover, determining the demand for these admixtures for various mixture designs could also be an issue for many end‐users. The use of retarders cannot be generally implemented as these chemicals usually lead to lower mechanical strengths at 1 day of hydration, which is already a key problem for these binders. Thirdly, the optimum water‐to‐binder ratio for LC^3^‐50 binders is not a straightforward feature. It is known that the water demand of LC^3^‐50 mortars is larger than those of their counterparts based on neat PC, due to the small size and large surface area of activated clay particles. However, this can be, and is being, corrected with PCE‐SPs. Many studies on pastes, mortars, and concretes have been reported with w/b = 0.50 to build knowledge following the standards [63, 127, 166, 185, 186, 187]. Other approaches have employed a smaller w/b ratio, i.e., 0.40, which result in lower porosities and higher compressive strengths [182, 188, 189, 190]. There are some publications with intermediate values, i.e., ~0.45–0.48 [191, 192, 193], which study selected changes of performances as a function of the w/b ratio [20, 174, 194, 195]. It is clear that lower w/b ratios lead to higher compressive strengths although there is a report that does not follow this trend [195]. In any case, in order to allow the pozzolanic reaction to develop at later ages, enough free water must be present in the systems, as water is both a reactant and the transport medium. This is convoluted with the PC milled to lower particle sizes, which results in higher fineness, and a greater water consumption. Therefore, the optimum water/binder ratio to be used in LC^3^‐50 systems, which is likely target‐feature dependent, is unknown to the authors. However, it is clear that more studies are needed where the flow (at early ages) and the compressive strengths at late ages (i.e., 28–365 days) are investigated as a function of the water/binder ratio within the most useful range, i.e., 0.40–0.50. These studies should ideally be linked to (natural) carbonation resistance assays.

Next, we develop three other important challenges with the final objective being that these low‐carbon cements replace type I PCs for most, if not all, applications.

### 8.1 Challenges in Thermal Activation

Thermal activation of kaolinitic (1:1) clays is a mature field, with several available technologies for clay activation; however, flash and stationary soak calcination remain the most widely applied at industrial scale [102, 196]. Despite this maturity, several scientific knowledge gaps persist, including (i) the role of cooling rates on calcined clay reactivity; (ii) the effects of the heating atmosphere beyond color control; (iii) role of other species (mainly iron and iron‐containing phases) on the pozzolanic features of the TA kaolinitic clays; and (iv) design and implementation of other ways of calcination, such as microwave heating, electric or solar powered furnaces, hydrogen‐fuel, etc.

The thermal activation of other clays has been far less studied. We do not repeat here what has been discussed above, but much more research is needed in the TA of 2:1 and modulated phyllosilicate minerals, including smectites, micas, palygorskites, and sepiolites, see Figure 1. Moreover, most of these rocks have a relatively high humidity which, nowadays is associated with CO_2_ emissions, due to the energy required to dry them. Therefore, additional CO_2_ footprint‐focused approaches are needed to establish the embodied carbon of the resulting low‐carbon cements.

### 8.2 Challenges in Mechanical Activation

There are three key challenges for the optimization of the MA parameters, and the processing of the cementitious binders prepared with MA clays.

- To expand MA to pilot‐plant level. This is essential for implementation and there are very few attempts currently underway. So far, only small amounts are being fabricated, which enable the study of pastes and some work on mortars. However, several kilograms, of each test, are required to assay flow (slump, cone, etc.). Addressing this will also allow a move toward research into concretes.
- The optimization of MA experimental conditions to maximize the pozzolanic activity of the resulting materials is not straightforward. The MA of clays requires the optimization of the parameters involved in the process. As detailed above, the key parameters are type of mill and for the planetary ball mill: rotation speed, milling time, and the BPR mass ratio. Higher rotation speeds (250–500 rpm) and longer milling times (30–120 min) enhance amorphization and structural transformations, though long milling times may not pay back. These factors collectively influence the reactivity of the final clay [138, 150]. A common starting point for MA using a planetary ball is a BPR ratio of 25, velocity of 500 rpm, and grinding time of 20 min [150].
- Processing optimization of cementitious systems with larger amounts of MA clays.
  c.1) The possible increase in SP demand for MA clays because of the increased SSA. However, the morphological alterations in MCA kaolinite, muscovite, and smectite clays, including the formation of spheroidal smoother particles, contributes to improving workability by reducing the external surface area, which helps reduce water demand [52, 138, 146]. It is known that the viscosity of suspensions is influenced, among other factors, by particle shape. Suspensions containing spherical particles exhibit lower viscosity values, at any given solid loading, compared to those with platelet‐shaped or irregular particles [197, 198]. Mañosa et al. (2024) found that the rounded particles produced by MA of kaolinitic clays led to a reduction in the demand for PCE‐SP compared to the related LC^3^ produced with the TA clay; for a similar workability (with w/b = 0.40) [146]. These findings on LC^3^, based on the MA of kaolinitic clays, are particularly significant. There are very few studies about the flowability of mortars based on MA clays. Further research is needed to explore the relationship between MA and the rheological behaviour of the corresponding low‐carbon cements.
  c.2) Gradual loss of fluidity over time. To address this, tailored PCE‐SPs have been successfully employed for TA kaolinitic and bentonite clays [114, 168, 169]. However, the loss of fluidity of low‐carbon cements fabricated with MA clays is completely unexplored, to the best of the authors knowledge.

### 8.3 Challenges in Sulfate Optimization

The use of activated clays as SCMs requires an adequate amount of sulfate anions in the binder. If this is not the case, the early age hydration reactions of the clinker phases are altered leading to lower early‐age compressive strengths of the corresponding mortars or concretes. Therefore, the need for sulfate amount optimization in low‐carbon cements, in general, and LC^3^, in particular, is fully acknowledged [192, 199, 200, 201, 202, 203, 204, 205].

However, how to define and obtain an optimum sulfate content is far from straightforward for the four main reasons detailed just below. First, a technique should be chosen and many approaches use isothermal calorimetry (mainly of pastes) [202, 203] with the figure of merit being the time separation between the (main) alite peak and the aluminate peak. This approach has the advantage of using smaller amounts of the sample. However, this procedure does not correlate well with the key property, i.e., early age compressive strengths of mortars. Therefore, we advocate here for using compressive strengths in mortars and complementing the results with isothermal calorimetry measurements in pastes and mortars. Second, even after this selection, another possibility opens up. This optimization can be carried out at 2 days of hydration (cement manufacture approach) [205] or at 1 day (concrete fabrication approach) [167]. We advocate here for 1‐day measurements as concrete is the final product. Thirdly, a water/binder ratio must be chosen and the results do not need to be the same for varying w/b ratios. Again w/b = 0.50 is mainly followed by cement‐oriented research and w/b = 0.40 is more common in concrete‐oriented investigations. We do not have any clear advice here but we carry out this optimization at w/b = 0.40. Fourthly and finally, this can be carried out for the mortars without admixtures (at w/b = 0.50) or with admixtures (w/b = 0.40, adding an SP to have similar flows, and SEA, if required). It is noted that common admixtures, like PCE‐SPs, require higher quantities of sulfates to properly function. We advocate here for optimizing the amount of sulfates for the final mortar [with the required amount of PCE‐SP, with SEA if needed, and at mortar relevant w/b ratio, likely to be 0.40–0.45). One example is given here to illustrate the importance and complexity of sulfate optimization. A binder with the composition 52%PC‐30%SCC‐15%LS‐3%GYP gave mortars with 13 and 58 MPa at 1 and 28 days respectively (SCC: smectitic calcined clay, w/b = 0.40; 0.8% by‐the‐weight‐of binder (bwb) of PCE‐SP to have a slump value (i.e., self‐flow) of 197(1) mm) [114]. This binder was oversulfated for a constant gypsum content along a series. The optimally sulfated binder, 52%PC‐31%SCC‐15%LS‐2%GYP gave mortars with 17 and 70 MPa at 1 and 28 days respectively (w/b = 0.40; 1.0% bwb of PCE‐SP to have of 191(3) mm). A lower amount of sulfates resulted in an increase of 31% of the compressive strength at 1 day and 21% at 28 days, but also a higher requirement of SP, i.e., 25% increase, to have similar flowability. Here, we do not treat other variables like fineness of the blend, curing temperature, accelerator addition, retarder addition, etc.

With the previous four choices in mind, it is not surprising that sulfate optimization is a hot research topic, without consensus. It is clear that calcined kaolinites require more sulfates than other activated clays which are poorer in aluminium. The LC^3^‐50 dosage 50/30/15/5, i.e., 50 wt.% of Portland clinker and 5 wt.% of gypsum, is a good starting point for kaolinite‐based LC^3^‐50 but not for similar materials based on montmorillonite, to cite just one example.

Due to the complexity of this multifactorial optimization, we advocate here for LC^3^‐50 binders to have an SO_3_ (or equivalent sulfate) content low, i.e., ∼2.0 wt.% (which could be slightly higher for kaolinite‐based ones). Then, the optimization of the sulfate, or SO_3_, content should be carried out like any other admixture (for instance the amount of PCE‐SP) following the procedure sketched above. Undoubtedly, much more robust research is needed for sulfate optimization for LC^3^‐50 based on different activated clays with varying pozzolanic reactivities.

## 9 Concluding Remarks

**FIGURE 9:**
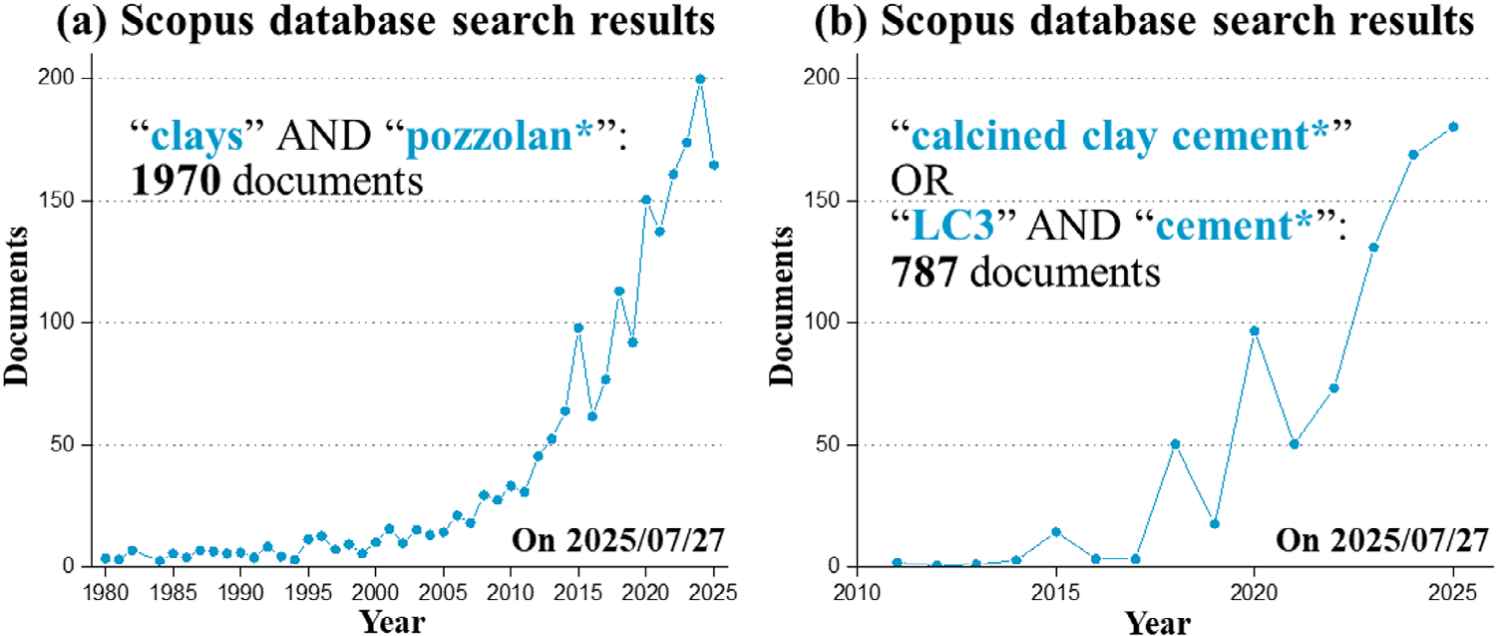
Results of two searches in the Scopus database (Elsevier) within <Article title, Abstract, Keyword> field. (a) Using the strings “clays” AND “pozzolan*”. (b) Using the strings “calcined clay cement*” OR “LC3” AND “cement*”.

Whereas Roman concretes [206], also called *opus caementicium*, are artificial stones prepared more than 2000 years ago by well‐designed mixtures of natural pozzolans, lime and water, natural pozzolans, in contrast, are silicates or aluminosilicates, generally of volcanic origin, that when milled to fine particles, react with calcium hydroxide in the presence of water to yield materials with cementitious properties [45]. As Vincent Thiéry [41], recently pointed out, calcined clays are not a new development but rather they have been used as artificial pozzolans since the 18th century, thanks to the pioneering works of Chaptal and Vicat. This is illustrated in Figure 9a, where it is shown that clays had been studied and used as pozzolans for many decades [207, 208], but with only moderate interest from the cement research community despite their widespread presence. This changed with the irruption of the LC^3^ project [209], led by Karen Scrivener, and the seminal publications of this consortium [51, 63, 210, 211]. This can be seen in Figure 9b, where it is shown that a sharp interest appeared around 2018 when the community was made aware of the potential of blends produced with Portland cements and a mixture of calcined and finely milled clays and limestone of high fineness.

As stated above, these low‐carbon cements contain: (i) Portland clinker, (ii) activated clay, (iii) limestone, and (iv) calcium sulfate. The most common formulation is the so‐called LC^3^‐50, where 15 wt.% of limestone is employed. The synergistic benefit of limestone in the improvement of hydration kinetics of clinker and calcined clays, through the provision of additional nucleation sites and the enhancement of aluminate reactivity by carboaluminate formation, is well documented. These carboaluminates, see reaction (4) as an example of hemicarboaluminate formation in addition to C‐A‐S‐H gel, further refine the porosity [189, 212] improving the mechanical strengths and decreasing the permeabilities, which is also very beneficial for durability performance. It is also known from mass balance calculations that only a relatively small fraction of limestone, less than 2 wt.% out of the 15 wt.% added, is consumed in carboaluminate formation. However, its role in nucleation sites and also filling empty spaces should not be overlooked. In fact, low‐hardness limestone is the most cost‐efficient way to add a harmless low‐solubility inorganic component with small particle sizes, i.e., a filler, to mortars and concretes with the additional advantage of its surfaces being very appropriated to induce a homogeneous arrangement of heterogeneously‐nucleated C‐S‐H gel.

This study has focused on activated clays but the role of other components must be taken into account in order to understand the chemistry and predict the performances of the resulting products: mortars and concretes. Although a generic composition is emerging 50/30/15/5, this does not mean that this composition is optimum for most applications. Depending on the figure of merit to be used: (a) overall price, (b) CO_2_‐footprint, (c) mechanical performances, (d) durability performances, different compositions could be chosen. Moreover, with the evolution of the standards from prescriptive to performance‐based, more choices will be available in the near future. This study has just been related to cement specifications but curing conditions may also evolve from standard water‐curing to more elaborate approaches including water‐precuring and CO_2_‐curing. The roles of admixtures in low‐carbon cement hydration is another very hot topic, and the chemical activation during water curing with tailored admixtures has not been discussed in detail due to space limitations [177]. Here, the mechanistic understanding of cement hydration by X‐ray micro‐and nano‐imaging [172] could play an important role, as compressive strengths at 1 day of curing must be enhanced to meet current practices for formwork stripping.

Given the decline in slag and fly ash production, and the expected sustained use of cement in construction worldwide over the coming decades, activated clays are becoming a key element in helping to reduce the carbon footprint of building materials. Much more collaborative research, reporting the results in a complete and structured way, is needed to enable the sustainable use of locally available clay.

## Funding

This work was supported by PID2020‐114650RB‐I00 and PID2024‐161797OB‐I00 grants from Agencia Estatal de Investigación, Spain, co‐funded by ERDF and Grant 101139298, acronym: syn4cem, call ERC‐AdG, from the European Union.

## Conflicts of Interest

The authors declare no conflicts of interest.

## Data Availability

The authors have nothing to report.
